# Deep learning and optical coherence tomography in glaucoma: Bridging the diagnostic gap on structural imaging

**DOI:** 10.3389/fopht.2022.937205

**Published:** 2022-09-21

**Authors:** Atalie C. Thompson, Aurelio Falconi, Rebecca M. Sappington

**Affiliations:** ^1^ Department of Surgical Ophthalmology, Wake Forest School of Medicine, Winston Salem, NC, United States; ^2^ Department of Internal Medicine, Gerontology, and Geriatric Medicine, Wake Forest School of Medicine, Winston Salem, NC, United States; ^3^ Wake Forest School of Medicine, Winston Salem, NC, United States; ^4^ Department of Neurobiology and Anatomy, Wake Forest School of Medicine, Winston Salem, NC, United States

**Keywords:** deep learning, artificial intelligence, glaucoma, optical coherence tomography, aging, basic sciences

## Abstract

Glaucoma is a leading cause of progressive blindness and visual impairment worldwide. Microstructural evidence of glaucomatous damage to the optic nerve head and associated tissues can be visualized using optical coherence tomography (OCT). In recent years, development of novel deep learning (DL) algorithms has led to innovative advances and improvements in automated detection of glaucomatous damage and progression on OCT imaging. DL algorithms have also been trained utilizing OCT data to improve detection of glaucomatous damage on fundus photography, thus improving the potential utility of color photos which can be more easily collected in a wider range of clinical and screening settings. This review highlights ten years of contributions to glaucoma detection through advances in deep learning models trained utilizing OCT structural data and posits future directions for translation of these discoveries into the field of aging and the basic sciences.

## Introduction

Glaucoma is a progressive optic neuropathy wherein retinal ganglion cell and retinal nerve fiber layer loss from optic nerve atrophy results in characteristic patterns of visual field loss (1). More than 76 million people were affected by glaucoma as of 2020, and it is projected to impact more than 111.8 million people by 2040, making it the most common cause of irreversible blindness worldwide (2). The only known modifiable risk factor for glaucoma is elevated intraocular pressure (IOP) (3), which increases the risk of subsequent vision loss if left untreated (4). However, despite the availability of effective treatments to lower IOP and thus slow down the rate of disease progression, a majority of patients with glaucoma are unaware they have the disease until it is advanced since the early stages are relatively asymptomatic (5). Thus, there is increasing interest in improving diagnostic and screening technologies so that glaucoma can be detected and treated at an early stage before the onset of irreversible blindness.

A number of diagnostic tests are employed in the clinical evaluation of glaucomatous optic neuropathy, including measurement of IOP and central corneal thickness, gonioscopy, visual field testing, fundus photography, and optical coherence tomography (OCT) (4). In recent years, however, OCT has risen to the forefront as the *de facto* diagnostic tool of choice for detecting the early onset of structural changes from glaucoma as well as its progression over time. Spectral domain (SDOCT) has excellent repeatability (6), and it is highly accurate even for detection of early lesions prior to the onset of visual field loss (7). SDOCT commercial software is not only able to segment and measure the thickness of the retinal nerve fiber layer (RNFL) and ganglion cell layer (GCL) (8), but also can be used to create 3-dimensional reconstructions of the optic nerve head (ONH), macula, and surrounding tissues. Measurements of the disc and rim area, the cup-to-disc ratio, and Bruch’s membrane opening-minimum rim width (BMO-MRW) can provide additional microstructural evidence of early glaucomatous damage.

Nevertheless, SDOCT is not currently recommended for population-based screening since the technology is expensive and requires skilled operators for image acquisition. Review of SDOCT imaging can also be complicated and time-consuming and clinicians can make errors when subjectively interpreting an array of different outputs produced by automated segmentation. The large number of varied parameters and plots produced by a single SDOCT test also increases the risk of committing a type 1 error or diagnosing glaucoma when it is not truly present. Moreover, over 40% of SDOCT may be affected by segmentation errors, which can lead to false positives and negatives (9–11). The risk of overdiagnosis and treating false positives is thought to outweigh the possible benefit of early detection (4). Distinction of glaucoma from age-related changes affecting the thickness of the retinal nerve fiber layer on SDOCT is also critical to the accurate detection of glaucomatous progression over time (12, 13). Furthermore, the different SDOCT platforms are not standardized, and no true reference standard exists, making comparison of output across platforms challenging (14). Thus, the gold-standard diagnosis for glaucoma has continued to be made on the basis of clinical judgement, combining clinical data from ophthalmic dilated fundus examination of the optic nerve head, intraocular pressure measurements, and interpretation of visual field and SDOCT tests.

The desire to develop novel technologies to improve ease, cost, and objectivity of glaucoma detection has spurred recent investigations into the development of artificial intelligence (AI) algorithms that can be applied to existing imaging data. Deep learning (DL) algorithms have shown particular utility in glaucoma detection given the ability of these convolutional neural networks (CNNs) to process complex ophthalmic imaging such as SDOCT of the optic nerve, macula, and anterior segment. Because DL algorithms can be trained to provide a single prediction about an image – i.e. whether it appears glaucomatous or normal – the algorithm can mitigate risk of committing a type 1 error and potentially decrease the time needed to review structural imaging in a clinical setting. Moreover, recent studies have demonstrated that DL algorithms can be trained using SDOCT to provide predictions about other structural imaging used in glaucoma such as fundus photographs. Application of DL algorithms have the potential to improve feasibility of glaucoma screening using low cost color fundus photography.

In this article, we will provide a brief overview of AI and DL before discussing some of the most recent advances in the development of DL algorithms trained with OCT imaging to assist with glaucoma detection on structural OCT imaging of the optic nerve, macula, and anterior segment, as well as glaucomatous damage on inexpensive color photography of the optic disc. Finally, we will highlight possible gaps in the field of DL as it applies to SDOCT and glaucoma, which will be critical to address in future translational work, as well as propose some new directions into the fields of aging and basic science.

### What is deep learning?

AI encompasses an array of automated computer programs that can mimic intelligent behavior with minimal human input (15). Machine learning is the broadest category of AI and refers to a method of automated data analysis wherein the machine learning classifier (MLC) is presented with multiple relevant examples in order to train it to automate a task (16–19). In comparison to traditional statistical analysis, MLCs can handle larger and more complex datasets which has made them of increasing interest in this age of ‘big data’ and electronic health records. Before MLCs can be trained, however, human input is required to identify the relevant features that the MLC needs to learn. While numerous examples of traditional MLCs exist, some common examples include random forest, support vector machines, and independent component analysis.

DL is a more recent development in the field of AI which has emerged as computational capabilities have increased. DL has made possible analysis of more complex data with predictions that sometimes exceed that of humans. The convolutional neural networks (CNNs) utilized in DL are modeled after the human visual cortex with many layers of interconnected neurons, or “nodes”, capable of autonomously processing and learning features from a training dataset (16). As data is input and passed through the series of convolutional layers, each layer assigns a weight to the data before passing it on to the next neuron, and these weights are subsequently adjusted to develop a classification system (20). CNNs are well-equipped to process very complex data types including two- and three-dimensional imaging. Moreover, human input is not required to identify the relevant features that the model needs to learn. Rather the CNN automatically identifies which features are relevant during the training process (21).

This fact causes DL models to offer several advantages over traditional MLCs. First, the DL model may be more objective and less labor intensive than traditional MLCs since *a priori* feature identification is not needed. However, in supervised learning, the DL model still requires a reference “ground truth” designation to input data for classification. For example, if the DL algorithm is viewing an OCT scan, then the “ground truth” label of whether the scan demonstrates glaucoma or not will help the algorithm learn to distinguish between the two outcomes. Nevertheless, DL models may be able to identify novel features or patterns in the data that humans had not previously detected. In unsupervised learning, for example. the CNN views unlabeled data and discovers new patterns or relationships with no human input. However, unsupervised learning has been less commonly applied to OCT data in glaucoma.

One drawback of DL is that the decision making, or prediction of the DL model is entirely automated and highly complex, making it impossible to trace all of the individual decision-making steps made during model building. This has led to a widespread perception that DL predictions arise from a “black box” (22). Also, given the complexity of the computations performed, CNNs require extremely large datasets for training, which are not always available, especially for ophthalmic imaging. Techniques such as transfer learning have been able to circumvent some of the challenges posed by the need for large datasets. In transfer learning, existing CNNs that are trained to perform a simpler task on large datasets can be further trained to perform more sophisticated tasks on more complex datasets of limited size (23). Neural networks have also been used to develop additional sophisticated analytic techniques such as generative adversarial networks, variational autoencoders, and transformer models. Thus, with advances in computational power, application of transfer learning techniques, and access to larger imaging datasets for training, DL has become a powerful tool for development of new approaches to OCT interpretation in glaucoma.

## Methods

**Figure 1:**
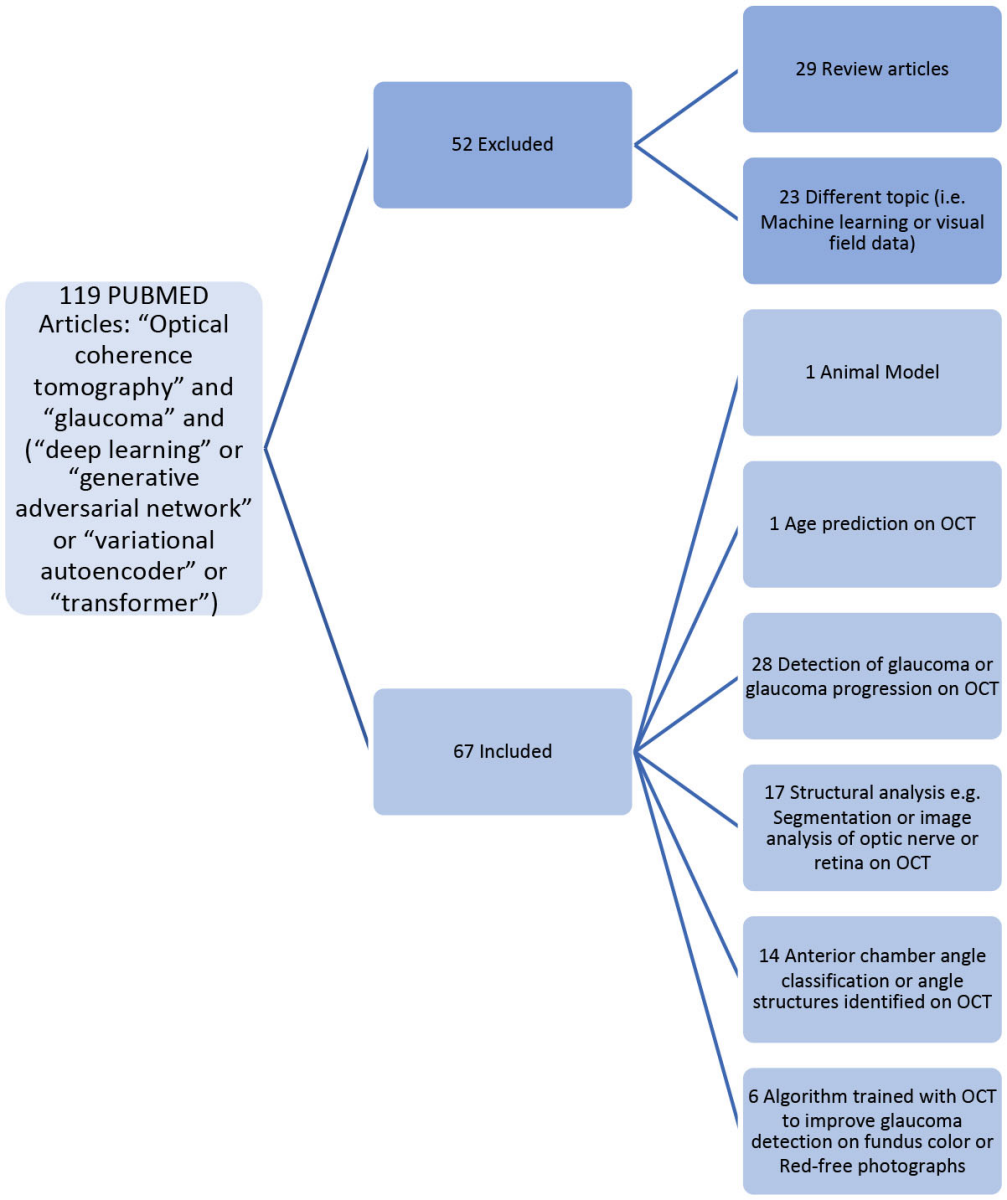
Flow diagram of articles reviewed.

Following the Preferred Reporting Items for Systematic reviews and Meta-Analysis (PRISMA), we conducted a systematic search of original studies wherein deep learning models were trained with OCT for detection of glaucoma on structural imaging. We searched PubMed using the terms “optical coherence tomography” AND “glaucoma” AND (“deep learning” or “generative adversarial networks” or “variational autoencoders” or “transformer”) for the date range of January 1, 2012, to January 1, 2022. This search yielded a total of 119 results from Pubmed (**Figure 1**). After two reviewers carefully read the articles, 29 were removed because they were review articles, and 23 were removed because the topic was not relevant (i.e. article on development of traditional machine learning classifier without deep learning techniques, algorithm developed for visual field analysis, etc.). In the final count there were 65 original research articles conducted in human subjects which presented results from deep learning models trained with OCT to either 1) improve detection of glaucoma or glaucoma progression on OCT (N=28) (**Supplementary Table 1**) (24–51), 2) improve structural analysis such as segmentation or image analysis of optic nerve and retinal microstructural elements relevant to glaucoma on OCT (N=17) (**Supplementary Table 1**) (52–68), 3) improve angle classification on anterior segment or swept source OCT (N=14) (**Supplementary Table 2**) (69–82), or 4) improve glaucoma detection on photos of the retina or optic nerve (N=6) (**Supplementary Table 3**) (83–88). An article about a DL algorithm trained to predict age from OCT RNFL was also reserved for discussion of the implications for aging research (89). In addition, an article about a DL model developed in rodent models of glaucoma was retained and combined with three other articles about application of DL in the basic sciences for the discussion of future directions (90–93).

### Data extraction

**Figure 2:**
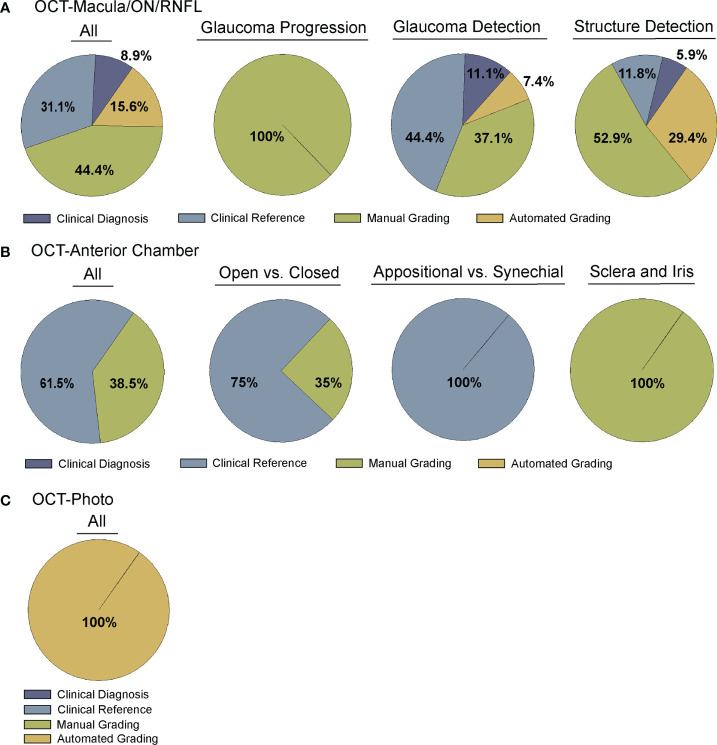
Comparison of Ground truth for deep learning algorithms. Studies were divided into four categories for ground truth, including clinical diagnosis, clinical reference, manual grading, and automated grading. **(A)** Comparison of all (far left) or sub-categorized (remaining) studies training deep learning algorithms for OCT of the optic nerve, RNFL, or macula (**Supplementary Table 1**), **(B)** Comparison of all (far left) or sub-categorized (remaining) studies training deep learning algorithms for OCT of anterior chamber anatomy (**Supplementary Table 2**), and **(C)** Comparison across all studies training deep learning algorithms for OCT – Photo pairs (**Supplementary Table 3**).

**Figure 3:**
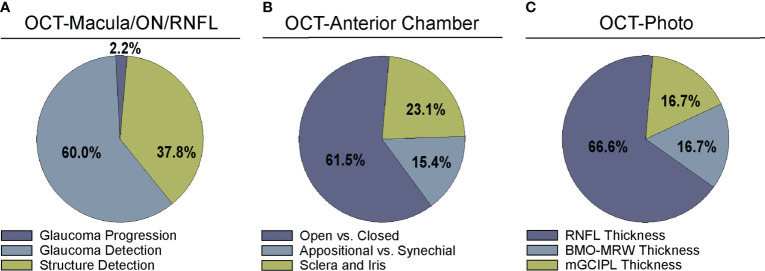
Comparison of algorithm output for deep learning algorithms. Studies were divided into categories based on output parameters for **(A)** studies examining OCT of the optic nerve, RNFL, or macula (**Supplementary Table 1**), **(B)** OCT of anterior chamber anatomy (**Supplementary Table 2**), and **(C)** OCT – Photo pairs (**Supplementary Table 3**).

**Figure 4:**
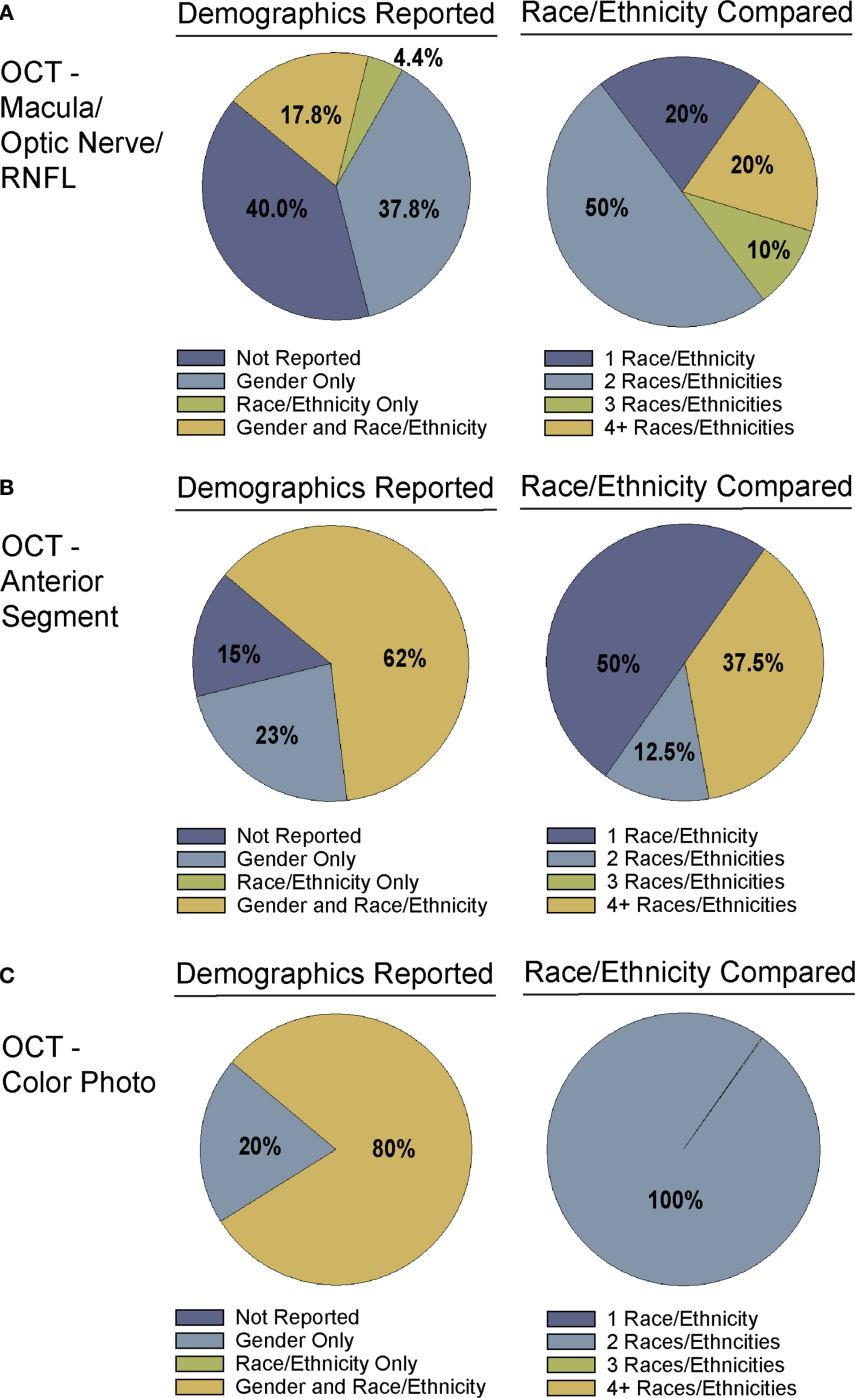
Demographic reporting of gender and race/ethnicity in algorithm testing and training. Studies for training and testing of deep learning algorithms applied to: **(A)** OCT of the optic nerve, RNFL, or macular (**Supplementary Table 1**), **(B)** OCT of the anterior chamber (**Supplementary Table 2**), and **(C)** OCT-Photo pairs (**Supplementary Table 3**) were categorized by reporting of gender and race/ethnicity (left). Studies reporting race/ethnicity were further categorized by the number of race/ethnicities compared and/or reported.

**Figure 5:**
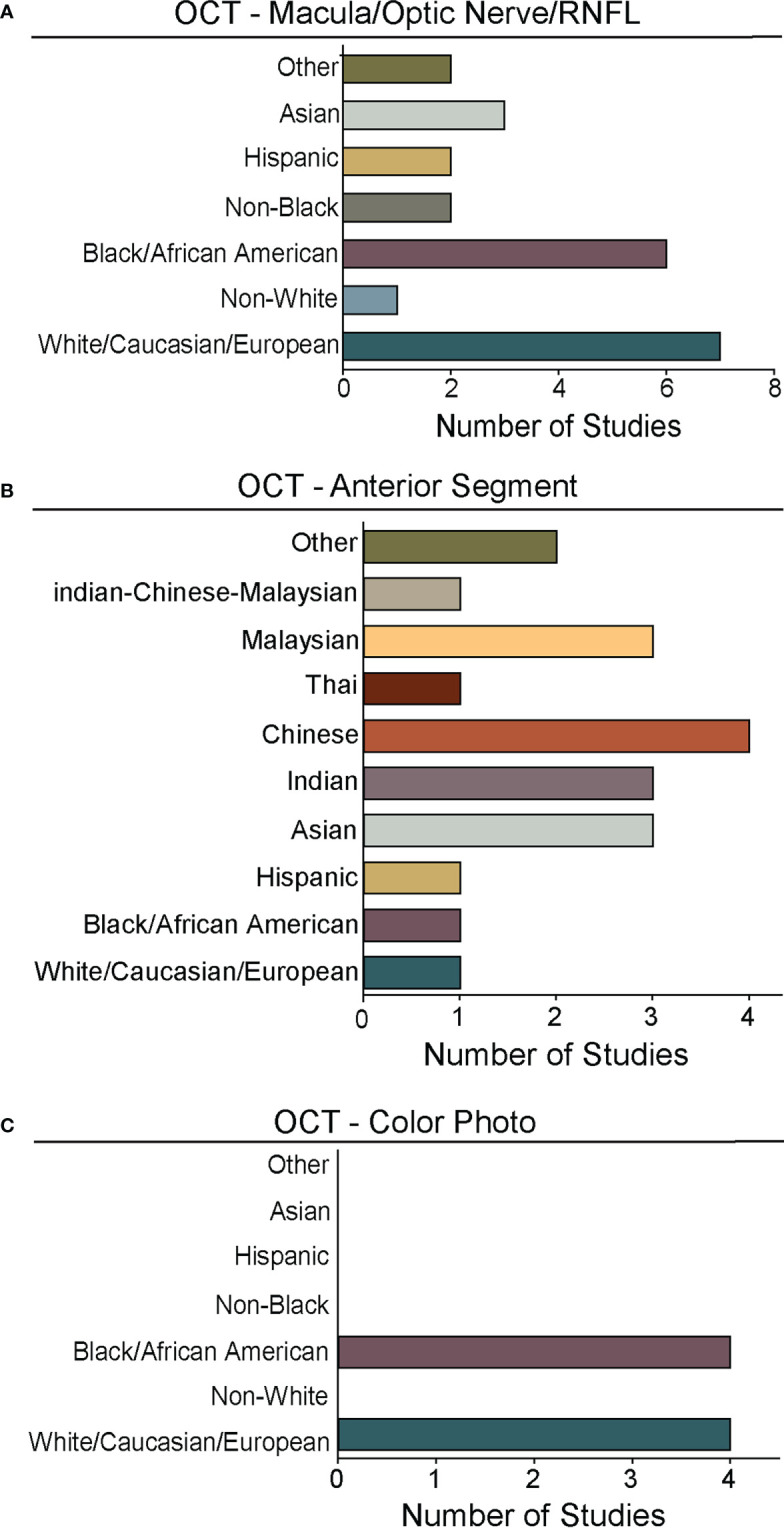
Races and ethnicities represented in algorithm testing and training. For studies from **Figure 4** that reported race/ethnicity, we graphed the number studies reporting data for specific races/ethnicities. As in **Figures 2**, **4**, we segregated studies that trained and tested deep learning algorithms for: **(A)** OCT of the optic nerve, RNFL, or macular (**Supplementary Table 1**), **(B)** OCT of the anterior chamber (**Supplementary Table 2**), and **(C)** OCT-Photo pairs (**Supplementary Table 3**). For ease of comparison, color coding for individual races/ethnicities is consistent in **A-C**.

The following data were extracted from the papers included in this review and are summarized in **Supplementary Tables 1-3** and **Figures 2**–**5**:

- Bibliographic information, i.e. authors, year of publication, journal
- Input for training the CNN, i.e. type of OCT or derived parameters or maps
- Ground truth or reference standard used for training the DL model (**Figure 2**)
- Primary output by DL algorithm, i.e. classification of glaucoma vs. normal, prediction of RNFL thickness, etc. (**Figure 3**)
- Training and test dataset characteristics including number or percentage of images in each set and diagnoses (e.g. glaucoma, suspect, normal)
- Demographic information, e.g. race/ethnicity, gender (**Figures 4**, **5**)
- Primary outcome of the study, e.g. reporting area under the curve (AUC) for discriminating between glaucomatous and normal eyes
- Results of any direct comparison of the results of DL versus traditional machine learning methods or automated parameters, if also conducted

## Results

### Deep learning models trained to detect glaucomatous damage on OCT

A majority of studies trained their DL model with OCT parameters or OCT imaging to diagnose glaucomatous structural damage on OCT (24–51) or identify structural components of the optic nerve or macula affected by glaucoma (52–68). Included articles used macular data (25, 43, 65, 68), optic nerve or RNFL scans and measurements (25–27, 29–32, 36, 39–42, 44, 46, 49–57, 59–63, 66, 67, 88), or a combination of OCT data from different types of scans or thickness maps, with or without additional clinical criteria (24, 25, 27, 28, 33–35, 38, 45, 47, 48, 61, 64) for training the DL algorithm (**Supplementary Table 1**). The ground truth used for training the DL algorithm varied. We classified the studies into four categories for ground truth. These categories were defined as studies utilizing: 1) a defined set of clinical parameters (clinical reference), 2) a known clinical diagnosis of glaucoma (clinical diagnosis), 3) grading of OCT images by trained personnel or clinical expert (manual grading), and 4) grading of OCT images by automated parameters (automated grading). We found that most studies utilized manual grading of OCT images (44.4%), followed by clinical reference (31.1%). Fewer studies relied upon automated parameters (15.6%) or clinical diagnosis (8.9%) as a reference standard (**Figure 2A**). Twenty-seven studies (60%) trained the DL algorithm to detect glaucoma, 17 studies (37.8%) focused on structural analysis, and one study (2.2%) identified glaucoma progression on OCT (**Supplementary Table 1**; **Figure 3A**).

Moreover, a majority of these studies compared the performance of DL models with other approaches such as traditional machine learning classifiers, values from automated segmentation, or hand-crafted features derived from OCT, with stronger performance by the DL model (25–27, 31, 34–36, 38, 44, 45, 47, 51, 66, 67). For example, Zheng et al. reported that their deep learning algorithm (AUC 0.99) performed significantly better than hand-crafted features from peripapillary RNFL images (AUC 0.895 for average hand-crafted feature) when detecting glaucoma on OCT (51). Lee and colleagues trained an ensemble DL model with a combination of GCIPL and RNFL thickness and deviation maps, resulting in an excellent AUC of 0.99 for distinguishing glaucoma from normal (35). This model outperformed individual OCT parameters derived by traditional automated segmentation, MLC, or visual field parameters.

Several groups also demonstrated that DL was particularly advantageous in detection of early glaucomatous damage on OCT before the onset of visual field loss. Asaoka and colleagues, developed a DL model to distinguish early glaucoma from normal eyes using OCT macular data acquired in an 8x8 grid (25). Since their OCT macular training dataset only had 178 images, they applied transfer learning techniques and built on a previously trained CNN. The DL AUC was 0.937 which was significantly greater than two other MLC models trained with similar data (AUC 0.82 for support vector model; AUC 0.674 for random forest model). Another advantage of their DL algorithm was that it performed well even though the training and test datasets were constructed with images from different OCT machines. Another DL algorithm trained with both RNFL and ganglion cell inner plexiform layer (GCIPL) thickness map data showed greater diagnostic ability for discriminating patients with glaucoma (AUC 0.957) or early-stage glaucoma (AUC 0.869) from normal subjects compared to a DL algorithm trained with either RNFL or GCIPL alone (47).

Distinction of early glaucoma from glaucoma suspects can also be particularly challenging, especially in patients who have “normal” intraocular pressure. For this reason, Seo and colleagues trained a DL algorithm to distinguish between glaucoma suspects and early normal tension glaucoma (NTG) using the Bruch’s membrane opening-minimum rim width (BMO-MRW) from OCT, a relatively new parameter that may be more accurate for detecting neuroretinal rim damage (44). Although the DL algorithm trained with BMO-MRW (AUC 0.959) performed better than the model trained with RNFL alone (AUC 0.914), the best performance occurred when combining three parameters – BMO-MRW, RNFL thickness, and RNFL color code classification (AUC 0.966). This DL algorithm trained with three parameters also outperformed several other machine learning models for early NTG detection (AUC 0.927-0.947). Thus, DL models trained with combinations of OCT parameter inputs showed particularly robust diagnostic ability compared to MLCs or algorithms trained with single parameters, especially in detection of early glaucoma.

In addition to traditional DL, hybrid models that combine DL with other approaches sometimes provided superior results compared to using DL alone or relying on manual segmentation. Muhammad et al. trained a CNN to extract features from swept-source OCT, and then trained a random forest classifier to predict glaucoma with those features derived from the resulting maps (38). This “hybrid DL” model was most accurate when trained using the RNFL probability map (93.1%) and outperformed the accuracy of parameters from traditional OCT segmentation or 24-2 and 10-2 Humphrey visual field (66.7% - 87.3%). Garcia and colleagues have also recently developed a novel DL methodology for diagnosing glaucoma on 3D SDOCT B-scans of the optic nerve (30). In their study, they first trained and validated a slide level feature extractor using 2-dimensional circumpapillary B-scans, and then combined the feature dependencies in the latent space *via* Long-Short-Term Memory (LSTM) networks with a CNN to improve glaucoma prediction from -3D optic nerve volume scans. Their end-to-end algorithm had relatively high accuracy (AUC 0.8847) for detecting glaucoma on a 3D SDOCT of the optic nerve. Another study by Raja and colleagues developed a hybrid DL framework to detect glaucoma on the SDOCT scans of the optic nerve head using the retinal ganglion cell profiles (ganglion cell complex, RNFL, ganglion cell-inner plexiform layer) (41). RAG-Net_v2_ was trained to extract the three features and the trained weights were used to classify glaucoma versus healthy with an AUC of 0.9871 which was higher than five other state-of-the-art solutions. Moreover, the hybrid framework was better able to distinguish between early and advanced glaucoma on OCT than RNFL thickness (accuracy=0.9117 vs. 0.7647) which may suggest it has potential application for detection of glaucoma progression. However, the study did not apply the hybrid system to a longitudinal dataset of OCT scans to assess progression.

Diagnosis of glaucoma progression over time on OCT is challenging since normal aging can also cause microstructural changes to the retinal nerve fiber layer over time (12, 13). However, DL may be able to more accurately detect progression than traditionally segmented parameters because the algorithm may draw on additional information when assessing the OCT image. For example, Bowd et al. compared the circumpapillary RNFL (cpRNFL) thickness from automated segmentation to eye-specific OCT RNFL-based region-of-interest (ROI) maps developed using unsupervised deep-learning auto-encoders (DL-AE) for the detection of glaucomatous progression (77). They found that cpRNFL-based region of interest maps developed using unsupervised DL-AE were more sensitive (0.90 vs. 0.63) and had similar specificity (0.92 vs. 0.93) for glaucoma progression compared to cpRNFL thickness. Moreover, the DL-AE ROIs had significantly steeper slopes for change over time compared to cpRNFL thickness values in both the progressing (-1.28 micons/year vs. -0.83 microns/year) and non-progressing eyes (-1.03 microns/year vs. -0.78 microns/year) suggesting that the DL-AE may be gleaning additional information beyond cpRNFL *via* the ROIs which may be important to glaucoma progression.

### Deep learning models trained to detect angle anatomy on OCT of the anterior segment

In clinic, gonioscopy is the gold standard for evaluating whether the anterior chamber angle (ACA) is closed or open, which is critical since angle closure places one at risk for acute or chronic angle closure glaucoma. However, gonioscopy is semi-subjective, and can only be performed through direct patient contact by an expert examiner. Contact-free anterior segment-OCT (AS-OCT) is able to capture images of the anterior chamber angle anatomy, and thus may be useful for angle closure detection. However, most current automated classification systems for AS-OCT provide only a binary classification of open versus closed without the ability to distinguish between subtypes of angle closure, and these systems are prone to misclassifications.

Fourteen articles have developed DL models that predicted the formation of the ACA on AS-OCT or swept source OCT with several comparing their performance against current automated systems (**Supplementary Table 2**) (69–82). Reference standards for training these DL algorithms ranged from gradings of ultrasound biomicroscopy images (73, 78) and gradings (69, 71, 74) of AS-OCT of the anterior chamber angle to clinical gonioscopy grades (75, 76, 79) (**Supplementary Table 2**; **Figure 2B**). Li and colleagues developed a DL algorithm able to distinguish between open versus narrow versus angle closure on AS-OCT with greater than 0.98 sensitivity and specificity, using ultrasound biomicroscopy as the reference-standard (73), while Xu et al.’s DL algorithm detected gonioscopic angle closure with an AUC of 0.928 on their test dataset (79). Fu et al. developed a DL model for distinguishing open from close angles on AS-OCT and compared its performance to that of an automated angle-closure detection system based on quantitative features (69). When using clinician gradings of the AS-OCT images as the reference standard, the DL model performed significantly better than the automated detection system (AUC 0.96 vs. 0.90, respectively). Fu et al. also developed a multilevel deep learning network (MLDN) which consisted of three parallel subnetworks that generated representations for multiple clinically relevant regions on the AS-OCT image (70). Their MLDN showed excellent performance for angle closure detection when tested on imaging from two different devices, the Carl Zeiss Visante AS-OCT (AUC 0.962) and Cirrus HD-OCT (AUC 0.952), and outperformed multiple deep learning networks.

While most articles demonstrated excellent detection of angle closure by DL, only three attempted to investigate more particular subtypes of angle formation (**Supplementary Table 2**; **Figure 3B**) (71, 72, 81). For example, early angle closure can be appositional, in which the trabecular meshwork is touching the cornea but without synechiae or scar tissue, whereas chronic angle closure is characterized by presence of synechiae. To address these different subtypes, Hao and colleagues trained a DL algorithm that could not only distinguish between open and closed angle formation, but also between appositional and synechial angle closure, with better performance (AUC 0.8005-0.8114) than conventional classifiers (AUC 0.6183-0.6648) in three different lighting settings (71).

In addition, three articles identified specific anatomic features such as the scleral spur or plateau iris on AS-OCT (**Supplementary Table 2**; **Figure 3B**) (74, 78, 80). Importantly, an investigation by Shen et al. identified biometric parameters such as iris curvature, lens vault, and angle opening distance, that may explain misclassifications of angle closure made by their OCT-based DL algorithm (77). Secondary angle closure can also occur due to particular iris formations, such as plateau iris, which can be challenging to diagnose in clinic. Wanichwecharungruang et al. demonstrated that DL can enhance detection of plateau iris formation on AS-OCT (78). Thus, DL algorithms show substantial promise for automating interpretation of AS-OCT for angle closure and subtypes of angle closure. In the future, incorporation of additional biometric elements may help refine the performance of these algorithms for angle closure detection.

### Deep learning models trained with OCT to detect glaucomatous damage on photos

One advantage of OCT over other structural imaging modalities such as fundus photographs is it provides more objective and reliable criteria for glaucoma detection than subjective human grades of photos. For this reason, six studies have utilized OCT data to serve as the ground truth when training DL algorithms to predict glaucomatous damage on fundus photos (**Figure 2C**) (83–88). In these studies, the algorithm learned to predict a structural parameter such as RNFL, BMO-MRW, or macular ganglion cell inner plexiform layer (mGCIPL) in datasets of imaging acquired in patients with glaucoma or healthy optic nerves (**Figure 3B**). Medeiros et al. was the first to propose this novel approach (87). He trained a “machine-to-machine” (M2M) deep learning algorithm with color optic disc photos labeled with the global RNFL thickness measurement from the corresponding SDOCT. When applied to optic disc photos, the M2M algorithm provided a prediction of RNFL thickness that was highly correlated with true RNFL from SDOCT (r=0.832, p<0.001), with MAE of 7.39 microns. In addition, the DL algorithm’s predicted RNFL discriminated between glaucomatous and normal eyes on color photos with similar accuracy to the true RNFL thickness from SDOCT (AUC 0.944 vs. 0.940, respectively). The performance of the M2M DL algorithm was subsequently compared to that of human graders for detecting which eyes had repeatable glaucomatous visual field loss (83). When applied to photos, the DL-predicted RNFL thickness was significantly more correlated with mean deviation from visual fields than the probability of glaucoma provided by human graders (rho=0.54 vs. 0.48, p<0.001). In a related paper, Thompson et al. paired color photos to the BMO-MRW thickness values from SDOCT to train a DL algorithm to quantify neuroretinal damage on the photos (88). When applied to color optic disc photos, the DL-predicted BMO-MRW was able to distinguish between glaucoma and normal eyes (AUC 0.945) with similar performance to actual BMO-MRW (AUC 0.933). Similarly, Lee and colleagues trained a hybrid DL model to predict the macular ganglion cell inner plexiform layer (mGCIPL) thickness when assessing a red-free retinal nerve fiber layer photograph by pairing the red free photograph with the SDOCT data (84). Their hybrid algorithm’s predicted mGCIPL thickness was highly correlated with the actual thickness (r=0.739, p<0.001) with an MAE of 4.76 microns. Moreover, the hybrid DL algorithm’s predictions showed excellent discrimination between eyes with glaucomatous visual field loss from healthy eyes when viewing the red-free photos (AUC 0.918).

Medeiros and colleagues have performed additional training of their M2M DL algorithm on a larger repository of SDOCT paired to fundus photographs collected on different cameras (Nidek and Visupac). They applied this model to a longitudinal cohort of color fundus photos and demonstrated that the DL RNFL predictions from the color photos could discriminate between eyes that were progressing and eyes that were not progressing (AUC 0.86), with even better performance for detecting fast progressors (AUC 0.96) (85). Moreover, their group has also shown that longitudinal changes in DL predictions of RNFL are able to predict conversion from glaucoma suspect to glaucoma. In the future, this overall approach of training DL algorithms to interpret fundus photographs while using OCT data for a reference standard could improve the utility of inexpensive fundus photography for detection of glaucoma and glaucoma progression, especially in low-resource settings.

## Discussion

Deep learning models show great potential for the accurate diagnosis of glaucoma on OCT without the need for human input. In some cases, such algorithms may even prove more accurate than either manual segmentation by human graders or output from automated segmentation in commercially available OCT software, especially for early glaucoma detection. Though further investigation is needed, at least one study suggested that DL may also be better able to predict glaucomatous progression on OCT than traditional linear regression of RNFL from automated segmentation (63). DL algorithms trained with OCT data may also be able to predict glaucomatous damage on other structural imaging (e.g. color fundus and red-free photos) and may even provide accurate predictions of glaucomatous progression on the photos over time. Thus, in the future, DL algorithms trained with OCT data may be able to improve the accuracy of low-cost photos for glaucoma detection in settings without access to OCT imaging.

Despite the exciting progress being made in the area of DL and glaucoma, a number of factors still limit the external generalizability of a majority of these DL models. Not all studies reported the demographic breakdown of their training or test datasets, and among those that did, the demographic variation was often limited to particular racial or ethnic groups (**Figures 4**, **5**). Similarly, some but not all studies reported the gender demographics of their study sample. For example, in the studies in **Supplementary Table 1** which trained and tested on OCT of the optic nerve or macula, 40% did not report on gender or race/ethnicity, 37.8% only reported on gender, 4.4% only on race/ethnicity, and 17.8% on both gender and race/ethnicity with a majority of patients being white/Caucasian or black/African American (**Figures 4A**, **5A**). By contrast, for the studies in **Supplementary Table 2** which focused on anterior chamber angle formation, 15% did not report on gender or race/ethnicity, 23% reported on gender only, and 62% reported on both, with most studies utilizing imaging acquired in Asian populations (**Figures 4B**, **5B**). For the studies in **Supplementary Table 3**, which used pairs of OCT and photos to train DL algorithms, 20% reported on gender only and 80% reported both gender and race, with patients being either white/Caucasian or black/African American (**Figures 4C**, **5C**). The differences in racial/ethnic breakdown are critical to consider since a CNN trained and tested in imaging acquired in a particular racial/ethnic group may not perform as well in other demographics who may have different optic nerve head or macular characteristics. For example, Asians tend to have more myopia which can result in tilted myopic discs and myopic degeneration, leading to segmentation errors on SDOCT that can mimic the appearance of glaucoma. Meanwhile larger optic disc size with physiologic cupping is more common in African Americans. Inclusion of OCT imaging across a range of racial/ethnic groups is critical when training DL algorithms not only because of these differences in optic nerve morphology, but also because glaucoma disproportionately affects racial minorities (39, 94, 95). However, in our review of the literature only a minority of studies reported on the racial demographics of their population (27, 30, 36, 37, 43, 57, 61, 66, 69, 74–76, 78, 79, 83–88). Maetschke et al. reported that 29.7% of included subjects were African American, 2.7% Asian, and 67.6% White (36). Similarly, Thompson et al. utilized an OCT dataset that was 21.7% African American (66). Asaoka et al. trained and tested their DL algorithms in OCT imaging acquired in Japanese patients (25). Meanwhile, Russakoff and colleagues trained their DL algorithm in a heterogenous sample of scans acquired in White, African American, and Asian participants (AUC 0.88), but the performance was lower when tested in an external dataset of Chinese patients (AUC 0.78) (43). Axial myopia may also impact SDOCT measurements leading to segmentation errors on OCT, but only one study directly addressed the influence of axial length on OCT measurements in glaucoma patients of Asian, African American, and White race (62). Olivas et al. was the only group to train and test their DL algorithm in Mexican patients (accuracy 86-90%) (39). Future studies should build more heterogeneous datasets for algorithm training and testing, as well as uniformly report the racial/ethnic and gender composition of their datasets.

Another challenge to real-world clinical implementation of many DL models is that the CNNs were trained and tested on idealized datasets and on imaging acquired in the same camera in the vast majority of cases. Thus, several recent studies have focused on the development of DL models that are capable of performing well in external datasets acquired in different cameras and in field data, where imaging may be affected by variability in quality. For example, Thakoor et al. created four different end-to-end DL models by employing fine-tuned transfer learning and also created a final CNN ensemble model (48). The accuracy of each of these DL models compared to earlier hybrid DL/MLCs was more robust in both laboratory and field test datasets, with smaller declines in performance when applied to field-collected datasets. Moreover, in one study by Devalla et al., a DL-based 3D segmentation framework was developed and shown to be applicable across data acquired in different OCT devices (54). This was accomplished by first pre-processing the images with a DL network “enhancer” that enhanced OCT image quality and harmonized image characteristics from the three devices, before training the DL algorithm “ONH-Net” to segment the OCT imaging acquired in each of the three different devices. In each case, the DL ONH-Net was able to segment ONH tissue from imaging acquired on a different device from the training dataset with excellent performance (Dice coefficient >0.92). These findings are also of particular interest since they demonstrated that DL models can be trained to accurately estimate specific features on SDOCT that are important to glaucoma diagnosis.

A significant limitation of the current literature is that a majority of studies trained and tested their DL algorithms using high quality OCT images and excluded those with concurrent retinal pathology. However, these DL algorithms designed for glaucoma detection may fail if applied in real-world settings, where glaucoma patients can have multiple comorbid retinal pathologies on OCT. Traditional automated segmentation of SDOCT images using the machine’s commercially available software has also been shown in numerous studies to be affected by segmentation artifacts (9–11), which may be due to vitreous traction, epiretinal membrane, or tilted disc from high myopia. Such artifacts can adversely impact interpretation of OCT studies leading to false positive and false negative results (81). Several groups have developed DL algorithms that can segment OCT optic nerve and macular tissue layers known to be impacted by glaucoma, and in some cases they evaluated performance in imaging impacted by artifacts (56). Yow et al. demonstrated that the predicted RNFL segmentation from a DL algorithm had similar accuracy for glaucoma detection compared to manual segmentation of the RNFL (67). Rezapour et al. trained a DL algorithm to measure a novel feature, peripapillary choroidal thickness, in glaucomatous eyes and compared eyes with axial myopia to those without myopia (62). Using manually segmented OCT B-scans as the ground truth or reference standard, they found that the DL algorithm provided high quality choroidal segmentation in over 95% of eyes within each myopia group – high myopia, mild myopia, and no myopia. A deep learning model used to segment the ganglion cell layer likewise performed better than manual segmentation (65). More recently, Mariottoni et al. trained a DL model to provide reliable segmentation-free estimates of RNFL thickness on SDOCT peripapillary circle B-scans, which proved advantageous in cases where the conventional automated segmentation algorithm had failed (61). Segmentation-free DL was able to predict RNFL thickness estimates with greater accuracy than automated segmentation, especially in poor quality images affected by segmentation errors. Thompson et al. subsequently demonstrated that this segmentation-free DL algorithm could be trained using the SDOCT circle scan to accurately distinguish between glaucoma and normal eyes (66). Moreover, the algorithm demonstrated a higher AUC for glaucoma diagnosis compared to the global RNFL thickness from automated segmentation (0.96 vs. 0.87) and was particularly sensitive in pre-perimetric glaucoma (0.92 vs. 0.93). DL algorithms can also be trained to detect segmentation errors on SDOCT imaging acquired in clinical practice. Jammal and colleagues developed a DL model that discriminated between OCT scans with and without segmentation errors (AUC of 0.979) and was 98.9% sensitive in scans with severe segmentation errors (57). Such an algorithm may help clinicians review and identify such errors on OCT imaging and thus mitigate the chance of an incorrect diagnosis. Successful removal of blood vessel shadow artifacts from OCT by algorithms like Deshadow GAN during preprocessing may also improve performance of other algorithms used in OCT segmentation (52).

### Future directions for deep learning applications in aging and the basic sciences

There is unprecedented interest in development of DL algorithms in glaucoma with many unexplored areas of investigation. Currently most studies have been developed using imaging acquired in older patients, with only one study dedicated to pediatric glaucoma (96). Moreover, since distinguishing between normal age-related and glaucomatous changes on OCT can be difficult, future studies should focus on whether DL can be harnessed to identify unique features associated with healthy aging. Current studies on deep learning and aging on OCT are limited but show promise. Shigueoka et al. used the whole circle SD-OCT B-scan image to train a CNN to predict patient age with a Mean Absolute Error (MAE) of 5.82 years (89). The DL model was also able to differentiate the youngest from the oldest subjects with an AUC of 0.962. Class activation maps showed that all layers were equally important in the DL algorithm, but the posterior vitreous seemed to be an important area for classification in the youngest group. The study further examined specific areas of the image to identify if they differed in predictive value for age compared to the entire B-scan. Analyzing the entire B-scan produced the lowest MAE when compared to SD-OCT individual structures of the vitreous, RNFL, retinal layers without the RNFL, and the choroid. The RNFL alone had the greatest MAE of 9.99 years. Similarly, Chueh et al. trained a DL algorithm to predict age from macular OCT with a MAE of 5.78; class activation maps likewise found that the whole layers of the retina, rather than the choroid, were important to the age predictions (97). Thus, both of these studies suggest that there are novel features or relationships between features on the full thickness retinal scan, rather than individual parameters like RNFL or choroid, that may be impacted by aging. Yow and colleagues trained a DL algorithm to segregate neuronal and vascular components within the cpRNFL on OCT/angiography and then compared correlations of RNFL thickness with age after excluding the vessels (98). They found that the ratio of major and micro-vessels to cpRNFL achieved a stronger correlation with aging (r=0.478, p<0.001) than the ratio of major vessels to cpRNFL (r=0.027, p=0.820). Exclusion of these blood vessels from the cpRNFL may improve measurement of the neuronal component which is important to detection of pathologic changes.

Another critical development in DL is the training of such algorithms for application in animal models. Imaging across various modalities provides a non-invasive tool for the detection and quantification of glaucoma pathology in animal models that provides streamlined translation to the same metrics in human patients (99). Despite the routine use of OCT, OCT-A, and fundoscopy in animal models, few studies to-date utilize AI approaches. In a recent study by Fuentes-Hurtado, et al. a DenseNet CNN was further trained to discriminate between glaucoma and healthy eyes in OCT imaging acquired in a rodent (rat) model, achieving an AUC of 0.99 (90). More recently, Choy et al. developed the first DL model to segment the Schlemm’s canal lumen on OCT in living mouse eyes (91). The budding use of AI in animal models of glaucoma is not limited to *in vivo* imaging. Two DL algorithms have been developed to quantify optic nerve pathology in rodent models of glaucoma (92, 93). The successful development and characterization of these algorithms indicates that studies utilizing animal models can meet the data requirements needed for DL. This raises the interesting notion that the translational impact of imaging metrics could be expanded to include DL algorithms. Can DL algorithms be both translated and reversed translated between animal models and humans? Future efforts in the development of DL algorithms that enable this translation, i.e. optimization of transfer learning potential, could be a vital next-step in AI for glaucoma diagnostics and management.

### Conclusions

Deep learning models trained with OCT data show great promise in detection of microstructural damage due to glaucoma and glaucoma progression over time. However, future studies will need to improve the generalizability of these models by training and validating these algorithms in different demographics. Moreover, the ability to detect glaucoma in the setting of comorbid retinal pathology and related imaging artifacts will be critical to their successful implementation. Development of DL algorithms in OCT acquired in animal models will also be pivotal to the success of drug development and other translational work across species.

## Data availability statement

Publicly available datasets were analyzed in this study. This data can be found here: https://pubmed.ncbi.nlm.nih.gov/?term=%22optical+coherence+tomography%22+and+%22glaucoma%22+and+%22deep+learning%22&filter=dates.2012%2F1%2F1-2022%2F1%2F1.

## Author contributions

Conceptual design (AT; RS); Data collection (AT; AF); Analysis or analytic review (AT; AF; RS); Manuscript primary draft (AT) and editing (AT; AF; RS*).* All authors contributed to manuscript revision, read, and approved the submitted version.

## Funding

AT receives support from the NEI K23EY030897. RS receives support from the NEI R01EY020496. The funders had no role in the design or preparation of this review article.

## Conflict of interest

The authors declare that the research was conducted in the absence of any commercial or financial relationships that could be construed as a potential conflict of interest.

## Publisher’s note

All claims expressed in this article are solely those of the authors and do not necessarily represent those of their affiliated organizations, or those of the publisher, the editors and the reviewers. Any product that may be evaluated in this article, or claim that may be made by its manufacturer, is not guaranteed or endorsed by the publisher.
