# Supplementary material for: Deep learning and optical coherence tomography in glaucoma: Bridging the diagnostic gap on structural imaging
Source: Front Ophthalmol (Lausanne). 2022 Sep 21;2:937205. doi: 10.3389/fopht.2022.937205 (PMC11182271; doi:10.3389/fopht.2022.937205)
Supplement: Supplementary file 1 [file Table_1.docx]

**Supplemental Tables**

**Table 1: Deep learning algorithms trained to interpret glaucomatous disease, progression or important anatomical features on OCT of the optic nerve or macula**

| **GLAUCOMA PROGRESSION** | | | | | | |
| --- | --- | --- | --- | --- | --- | --- |
| **Author, *Journal*, Year** | **Input used for training deep learning algorithm** | **Deep learning output** | **Ground Truth/Reference Standard** | **Datasets for training and testing** | **Dataset demographics** | **Main Study Finding(s)** |
| Bowd et al., *Transl Vis Sci Technol.*, 2021 | A deep learning autoencoder was trained using tissue thickness measurements from the high-resolution optic nerve head cube scan (SD-OCT) | Classified glaucoma progression vs. non-progression by the Deep learning-autoencoder (DL-AE) Regions of Interest (ROI) map | ROIs for likely progression of glaucoma determined by standardized review of serial optic disc stereophotographs | Progressing glaucoma: 44 eyes / 33 patients.  Non-progressing glaucoma: 303 eyes / 189 patients.  Healthy eyes: 109 eyes / 59 participants.  Stable glaucoma eyes: 50 eyes / 27 patients | Healthy: Female (70%).  Non-progressing:  Female (66%).  Progressing: Female (68%) | The sensitivity for detecting change in progressing eyes was greater for DL-AE ROIs than for global cpRNFL annulus thicknesses (0.90 and 0.63, respectively). The specificity for detecting not likely progression in non-progressing eyes was similar (0.92 and 0.93, respectively). The mean rates of change in DL-AE ROI were significantly faster than for cpRNFL annulus thickness in progressing eyes (−1.28 µm/y vs. −0.83 µm/y) and non-progressing eyes (−1.03 µm/y vs. −0.78 µm/y) |
|  | | | | | | |
| **GLAUCOMA DETECTION** | | | | | | |
| **Author, *Journal*, Year** | **Input used for training deep learning algorithm** | **Deep learning output** | **Ground Truth/Reference Standard** | **Datasets for training and testing** | **Dataset demographics** | **Main Study Finding(s)** |
| Shin et al., *Journal of Glaucoma*, 2021 | Trained two CNNs – fusion by convolutional network (FCN) and fully connected network (FFC) – using OCT wide-field report of images from SS-OCT | Predicted healthy vs. glaucomatous eyes; and predicted early stage glaucoma | Clinical diagnosis of normal vs. glaucoma based on clinical dilated exam, characteristics of optic disc, IOP, RNFL defects on red-free fundus imaging, and visual fields. | Overall: 675 eyes/images: 258 healthy, 417 glaucoma.  For binary classification - Training (56.6%; 382 images), 137 Normal, 245 Glaucoma.  Testing (43.4%; 293 images): 121 Normal, 172 Glaucoma.  For multi-level classification to detect early stage glaucoma – Training - 274 images; Testing - 217 images. | Overall –363 Male (53.8%),  Normal –145 Male (56.2%),  Glaucoma –218 Male (52.3%) | Fusion by convolutional network (FCN) achieved an area under the receiver operating characteristic curve (AUC) of 0.987 (95% confidence interval, CI: 0.968-0.996) and an accuracy of 95.22%. In contrast, Fusion by fully connected network (FFC) achieved an AUC of 0.987 (95% CI, 0.971-0.998) and an accuracy of 95.90%. Both FCN and FFC outperformed the conventional method (P<0.001). In detecting early glaucoma, both FCN and FFC achieved significantly higher AUC and accuracy than the conventional approach (P<0.001) |
| Bowd et al., *American Journal of Ophthalmology*, 2021 | VGG16 CNN was  trained using *en face* 4.5 x 4.5 mm radial peripapillary OCTA ONH images - Extracted circumpapillary RNFL, radial peripapillary capillary, whole image capillary, and vessel densities; Three Gradient Boosting Classifier (GBC) ensemble classifiers were also trained – 1) wiVD GBC included whole image, superior hemiretina, and inferior hemiretina vessel densities 2) wiCD GBC included whole image, superior hemiretina, and inferior hemiretina capillary densities 3) wiVD and CD GBC included whole image, superior hemiretina and inferior hemiretina vessel and capillary densities 4) cpRNFL GBC trained on cpRNFL global and quadrant measurements | Predicted healthy vs. glaucomatous eyes | Glaucoma diagnosis using graded stereodisc photos and visual fields | Overall: 130 eyes/ 80 healthy patients;  275 eyes/185 glaucoma patients.  Training: 230 eyes / 148 glaucoma patients. 102 eyes / 60 healthy individuals.  Testing: 45 eyes / 37 glaucoma patients. 28 eyes / 20 healthy patients | Healthy:  Female (72%).  Non-white (41%)  Glaucoma:  Female (51.5%).  Non-white (38%) | Adjusted areas under precision recall curves (AUPRCs) for GBC models were 0.89 (95% CI: 0.82, 0.92) for whole image vessel density GBC, 0.89 (0.83, 0.92) for whole image capillary density GBC, 0.91 (0.88, 0.93) for combined whole image vessel and whole image capillary density GBC, and 0.93 (0.91, 095) for RNFL thickness GBC. The adjusted  AUPRC using CNN analysis of *en face* vessel density images was  0.97 (0.95, 0.99) resulting in significantly improved classification compared to GBC OCT/A-based results and GBC OCT-based results (P ≤ 0.01 for all comparisons) |
| Lee et al., *Journal of Glaucoma*, 2020 | NASNet was trained with GCIPL thickness map, GCIPL deviation map, RNFL thickness map, and RNFL deviation map | Predicted glaucomatous or non-glaucomatous probability | Glaucoma diagnosis and grade vs. non-glaucoma using graded stereodisc photos, redfree images, and visual fields | 307 images / 282 eyes (47 eyes assigned to early glaucoma stage, 39 assigned to moderate to severe glaucoma stage).  Training (69.9%);  Testing (30.1%) | Nonglaucomatous: Male 80 (40.8%).  Glaucomatous:  Male 26 (30.2%) | DL achieved an AUC of 0.990 (95% CI: 0.975-1.000) with a sensitivity of 94.7% and a specificity of 100.0%, which was significantly larger than the AUCs with all of the optical coherence tomography and SAP parameters: 0.949 (95% CI: 0.921-0.976) with average GCIPL thickness (P=0.006), 0.938 (95% CI: 0.905-0.971) with average RNFL thickness (P=0.003), and 0.889 (0.844-0.934) with mean deviation of SAP (P<0.001; DeLong test) |
| Kim et al., *Journal of Clinical Medicine,*2020 | VGG-19 CNN was trained using deviation and thickness maps of the RNFL and GCIPL on SD-OCT | Predicted glaucoma vs. controls; and distinguished early vs. moderate vs. severe glaucoma | Primary open angle glaucoma diagnosis vs. controls diagnosis based on clinical data, IOP, gonioscopy, graded optic disc and RNFL photographs and visual fields | Training: 7,288 images / 1,822 eyes (332 control, 1490 glaucoma).  Internal validation: 1,700 images / 425 eyes (104 control, 321 glaucoma).  External validation: 1,420 images / 355 eyes (108 control, 247 glaucoma) | Training dataset: Control – Male (49.4%);  Glaucoma – Male (58.9%).  Internal Validation dataset:  Control – Male (42.9%);  Glaucoma – Male (59.2%);  External validation dataset:  Control - Male (59.3%); Glaucoma – Male (61.1%) | The glaucoma-diagnostic ability was highest when the deep learning system used the RNFL thickness map alone (AUC 0.987), followed by the RNFL deviation map (AUC 0.974) for training. For deep learning systems using combinations of OCT maps, the RNFL and GCIPL deviation maps demonstrated the highest diagnostic performance (AUC 0.979), followed by all four OCT maps (AUC 0.977), and RNFL and GCIPL thickness maps (AUC 0.964). |
| Zheng et al., *Graefe's Archive for Clinical and Experimental Ophthalmology*, 2020 | Inception-V3 was trained using SD-OCT images of pRNFL | Predicted glaucoma vs. normal | Glaucoma diagnosis vs. normal based on optic disc color photo and visual field grades | Training:  1501 images  -690 images from 153 glaucoma patients;  -811 images from 394 normal subjects.  Testing:  102 images  -50 images from 50 glaucoma patients;  -52 images from 52 normal subjects | Training:  Normal – Female (51.5%).  Glaucoma - Female (35.3%).  Testing:  Normal - Female (61.5%).  Glaucoma - Female (16%) | Compared the diagnostic capability between DL model and hand-crafted features (HCFs) of pRNFL parameters. The DL model achieved an AROC of 0.99 (95% CI: 0.97 to 1.00) which was significantly larger than the AROC values of all other HCFs |
| Asaoka et al., *AJO,*2019 | A deep learning CNN was trained using 8x8 grid macular RNFL thickness and ganglion cell complex layer thickness from spectral-domain OCT | Detected early glaucoma vs normal eyes | Diagnosis of glaucoma based on full ophthalmic exam and visual field testing | 4,316 images / 1371 eyes with open angle glaucoma / 193 normal eyes. Training (47.6%) and testing (52.4%) | Training:  Healthy – Female (44.0%).  Glaucoma - Female (62.8%).  Testing:  Glaucoma - Female (57.0%).  Healthy - Female (68.3%) | The AROC with the DL model was 93.7%. The AROC significantly decreased to between 76.6% and 78.8% without the pretraining process. Significantly smaller AROCs were obtained with random forests and support vector machine models (82.0% and 67.4%, respectively) |
| Muhammad et al., *Journal of Glaucoma,*2017 | A hybrid deep learning model was trained using widefield swept source OCT images including RGC thickness map, RNFL thickness map, RGC probability map, RFNL probability map, and *en face* projection | The CNN derived feature vectors which were subsequently used to train a random forest classifier to classify “healthy” and “glaucomatous” eyes. | Diagnosis of glaucoma or healthy based on fundus photos, chart information, OCT and visual fields | 102 eyes /102 patients  –  57 glaucomatous eyes; 45 healthy/suspects eyes | Not reported | The accuracy of the hybrid deep learning model ranged from 63.7% to 93.1% depending upon the input map. The retinal nerve fiber layer probability map had the best accuracy (93.1%), with 4 false positives, and 3 false negatives. In comparison, the accuracy of the OCT and 24-2 and 10-2 VF metrics ranged from 66.7% to 87.3% |
| Lee et al., *Br J Ophthalmol.,*2020 | NASnet was trained using GCIPL/RNFL thickness and deviation maps | Predicted glaucomatous optic neuropathy (GON) vs. compressive optic neuropathy (CON) | Diagnosis of GON or CON based on IOP, stereo disc and red free photos, and visual fields | 80 eyes / 80 patients with GON.  54 eyes / 54 patients with CON | CON: Males (46.3%).  GON: Males (30.0%) | The deep learning system achieved an AUC of 0.990 (95% CI: 0.982 to 0.999) with a sensitivity of 97.9% and a specificity of 92.6% in five-fold cross-validation testing, which was significantly larger than the AUCs with traditional parameters: 0.804 (95% CI: 0.737 to 0.872) with temporal raphe sign, 0.815 (95% CI: 0.734 to 0.896) with superonasal GCIPL and 0.776 (95% CI:b0.691 to 0.860) with superior GCIPL thicknesses (all p<0.001) |
| Ran et al., *Lancet Digital Health,*2019 | ResNet was trained using paired SD-OCT 3D volumes and 2D Line Scanning Ophthalmoscope *en face* images of ONH | Predicted glaucomatous optic neuropathy vs. no glaucomatous optic neuropathy | SDOCT images of RNFL graded as glaucomatous optic neuropathy (GON) or no GON based on defect that correlated with visual field loss | Training: 4877 volumes.  Training (60%), Testing (20%), and Primary Validation (20%).  Three independent datasets including 546, 267, and 1231 volumes, respectively, were used for external validation | Training, testing, and primary validation dataset:  GON – Male (53.5%);  No GON – Male (38.6%)  External validation dataset 1:  GON – Male (55.4%);  No GON – Male (61.8%);  External validation dataset 2:  GON – Male (46.6%);  no GON – Male (58.3%) | The 3D deep-learning system had an area under the receiver operation characteristics curve (AUROC) of 0.969 (95% CI: 0.960–0.976), sensitivity of 89% (95% CI: 83–93), specificity of 96% (92–99), and accuracy of 91% (89–93) in the primary validation, outperforming a two-dimensional deep-learning system that was trained on en face fundus images (AUROC 0.921 (95% CI: 0.905–0.937); p<0·0001). The 3D deep-learning system performed similarly in the external validation datasets, with AUROCs of 0.893–0.897, sensitivities of 78–90%, specificities of 79–86%, and accuracies of 80–86% |
| Thakoor et al., *IEEE Trans Biomed Eng*, 2021 | Five different hybrid deep learning/machine learning models and robust end-to-end DL models and robust CNN ensemble models were previously trained using OCT-derived images, including RNFL and RGCP probability maps and thickness maps as well as full OCT reports | Predicted early glaucoma vs. healthy | OCT expert grading of OCT images for glaucomatous vs. not glaucomatous damage | Training, validation, and testing were previously carried out in an approximately 55%:20%:25% split (395 images for training, 145 images for validation, and 197 images for testing). | Not reported | In this study, five previously trained Hybrid DL/ML models and end-to-end DL models were applied to a field test set of 135 OCT RNFL probability maps and to the original lab dataset. Both Hybrid/DL/ML models (range 94-95.7) and end-to-end DL models (range 90.4-97) had greater accuracy in lab data than field data (range 69.6-80.7) and (range 83-91.1, respectively). |
| Sun et al., *Br J Ophthalmol.*, 2021 | Dual-input CNN was trained using RNFL and macular GCIPL thickness maps | Predicted early-stage glaucoma vs. glaucoma vs normal | Primary open angle glaucoma vs control based on characteristic rim notching or RNFL defects on red-free photographs with or without visual field loss | The data set used in this study (training and testing) included 777 eyes of 777 subjects (462 eyes/462 subjects with POAG and 315 eyes/315 normal subjects).  Among the 462 POAG eyes, 219 were early-stage, and 243 were moderate-to-severe stage.  For the training data set, 621 image sets in total (252 normal, 174 early POAG, 195 moderate-to-severe POAG) were used.  During the testing, 156 independent image sets (63 normal, 45 early-stage glaucoma, 48 moderate-to-severe-stage POAG) were assessed by each of the deep-learning algorithms. | Healthy: Male (50.2%).  Early POAG: Male (55.3%).  Moderate-to-severe POAG: Male (53.5%) | For the test data set, the dual input convolutional neural network (DICNN) could distinguish between patients with glaucoma and normal subjects accurately (Accuracy 92.793%, AUC 0.957 (95% CI: 0.943 to 0.966), Sensitivity 0.896 (95% CI: 0.896 to 0.917), Specificity 0.952 (95% CI: 0.921 to 0.952)). For distinguishing between patients with early-stage glaucoma and normal subjects, DICNN’s diagnostic ability (Accuracy 85.185%, AUC 0.869 (95% CI: 0.825 to 0.879), Sensitivity 0.921 (95% CI: 0.813 to 0.905), Specificity 0.756 (95% CI: 0.610 to 0.790)) was higher than convolutional neural network algorithms that trained with RNFL or GCIPL separately. |
| Seo et al., *Sci Rep.,* 2020 | Deep neural network (DNN) was trained using BMO-MRW and RNFL thickness and color code from OCT images | Predicted early normal-tension glaucoma vs. glaucoma suspects | Diagnosed with normal tension glaucoma or glaucoma suspect based on clinical exam criteria including IOP, visual field defects, and RNFL defects on red-free imaging | 397 eyes / subjects (229 eyes/subjects were glaucoma suspects.  168 eyes/subjects were early normal-tension glaucoma.  Training (80%) and Testing (20%) | Glaucoma suspect: Female (54.18%).  Normal-tension glaucoma: Female (44.1%) | The deep neural network model (DNN) achieved highest diagnostic performance, with an AUC of 0.966 (95% CI: 0.929-1.00) in classifying either GS or early NTG, while AUCs of 0.927-0.947 were obtained by other machine-learning models. The performance of the DNN model considering all three OCT-based parameters was the highest (AUC 0.966) compared to the combinations of just two parameters. As a single parameter, BMO-MRW (0.959) performed better than RNFL alone (0.914). |
| Russakoff et al., *Transl Vis Sci Technol.*, 2020 | gNet3D CNN was trained using SD-OCT macular cubes | Predicted referable for glaucoma vs. non-referable | Referable for glaucoma (included true glaucoma, pre-perimetric glaucoma, and high-risk suspects) vs. non-referable for glaucoma based on grading of fundus photographs, visual fields, OCT reports, and clinical examinations, including IOP, and treatment history | 1095 eyes of 586 patients were obtained for training and primary validation. 667 eyes of 381 patients labeled as referable cases comprised of 514 eyes from 287 patients with a diagnosis of glaucoma, 41 eyes from 26 patients with a diagnosis of PPG, 112 eyes from 68 patients with a diagnosis of being high-risk glaucoma suspects, and a total of 428 eyes of 249 patients labeled as non-referable cases comprised of 320 eyes from 183 definitive normal patients, and 108 eyes from 66 low-risk glaucoma suspects. | Six datasets.  1: Asian (43.5%), White (38.6%), African-American (5.04%), Hispanic (9.14%).  2: Asian (48.60%), White (33.40%), African-American (6.40%), Hispanic (7.20%).  3-6: Asian (100%) | The AUC for the development dataset for distinguishing referable glaucoma was 0.88 for the CNN using homogenization, 0.82 without homogenization, and 0.81 for a CNN architecture from the existing literature. For the external validation datasets, which had different glaucoma definitions, the AUCs were 0.78 and 0.95, respectively |
| Díaz-Alemán et al., *Arch Soc Esp Oftalmol (Engl Ed),* 2021 | Trained two DL models (ResNet50 and VGG19) with three infrared images - confocal scan image of the fundus, cut-out of the first centered on the optic nerve, and SD-OCT image of the GCL | Predicted glaucoma vs. normal | Clinical diagnosis of glaucoma vs. normal | 498 eyes (312 are glaucoma and 186 are normal) / 298 patients.  Training and validation (80%) and testing (20%). | Glaucoma: Male (52.9%).  Healthy: Male (36.56%) | In the test set, the precision of the models was 96% (ResNet50) and 96% (VGG19) for the GCL images, 90% (ResNet50) and 90% (VGG19) for the optic nerve images and 82% (ResNet50) and 84% (VGG19) for the fundus images. The ROC area in the test was 0.96 (ResNet50) and 0.97 (VGG19) for the GCL images, 0.87 (ResNet50) and 0.88 (VGG19) for the optic nerve images, and 0.79 (ResNet50) and 0.81 (VGG19) for the fundus images |
| An et al., *Sci Rep.,* 2021 | Different flat and hierarchical classification models were trained using varying combinations of projection images, *en face* images, horizontal B-scan OCT images crossing the disc center, and vertical B-scan OCT images crossing the disc center | Predicted Glaucoma vs, healthy with a low-level model and classified glaucoma subtypes with a high-level model | Label of normal vs. glaucoma and subtype (focally ischemic (FI), myopic glaucomatous (MY), generalized enlargement (GE), and senile sclerotic (SS) discs) were provided by human graders reviewing the four types of swept source OCT images | A total of 156 normal, 118 FI, 266 GE, 307 MY, and 107 SS eye images were included.  Training (80%) and Testing (20%). | Not reported | The average weighted accuracy and Cohen’s kappa for three randomized test datasets were 0.839 and 0.809, respectively. |
| Sułot et al., *PLoS One.,* 2021 | A CNN was trained using ONH *en face* images, captured with scanning laser ophthalmoscopy (SLO) during standard OCT imaging of the posterior segment | Glaucoma vs. normal | Glaucoma vs. normal based on IOP, visual fields, OCT RNFL thickness and dilated stereoscopic examination of the ONH. | 227 SLO images of 227 subjects: 105 glaucoma and 122 controls | Not reported | The study shows that cross-validation deep learning ensemble based on SLO images achieved a good discrimination performance an accuracy of up to 0.962. |
| Maetschke et al., *PLoS One.*, 2019 | A CNN was trained using raw, unsegmented OCT volumes of the optic nerve head | Classified eyes as healthy vs. glaucomatous | Glaucoma vs. healthy based on presence of 2 glaucomatous consecutive visual field defects | 624 patients/1110 scans total – 137 patients/263 scans were healthy, 432 patients/847 scans were POAG.  The data set was split into 888 training scans (216 healthy eyes/672 POAG eyes), 112 validation scans (30 healthy eyes/82 POAG eyes), and 110 test scans (17 healthy eyes/93 POAG eyes) (80%, 10%, 10%). | Healthy: Female (64.23%), White (74.26%), Black (22.06%), Asian (3.68%).  POAG: Female (50.23%), White (65.70%), Black (31.82%), Asian (2.48%) | Logistic regression was found to be the best performing classical machine learning technique with an AUC of 0.89. However, the deep learning approach achieved a substantially higher AUC of 0.94. On class activation maps the neuroretinal rim, optic disc cupping, and lamina cribrosa regions were important for glaucoma detection |
| Wang et al., *Med Image Anal.*, 2020 | An end-to-end multitask learning CNN with ResNet18 was rained using B-scan OCT images | Predicted glaucoma vs. normal | OCT images labeled as glaucoma vs. normal | Hong Kong dataset: 975,400 B-scans / 4,877 volumes training and validating. Stanford dataset: 246,200 B-scans / 1,231 volumes testing | Not reported | The proposed method outperforms the baseline methods and two glaucoma experts by a large margin, achieving volume-level Area Under ROC Curve (AUC) of 0.977 on Hong Kong dataset and 0.933 on Stanford dataset, respectively |
| Panda et al., *Am J Ophthalmol.,* 2021 | A CNN was trained to segment 3 neural-tissue and 4 connective-tissue layers of the ONH; then segmented OCT were processed by an autoencoder network with a parallel branch for binary classification in a low-dimensional latent space (LS), whereas the decode and classification branches reconstructed the images and classified them as glaucoma or nonglaucoma. Principal components analysis on latent parameters was also performed. | Predicted glaucoma vs. non-glaucoma | Manual segmentation of OCT images | Our training, validation, and test sets had 5,290 (70.2%, nonglaucoma: 2791, glaucoma: 2499), 1,130 (15%, nonglaucoma: 742, glaucoma: 388), and 1,111 (14.8%, nonglaucoma: 720, glaucoma: 391) segmented OCT images, respectively.  Training (70%), Validation (15%), and Testing (15%) | Cohort 1: Male (49%).  Cohort 2: Male (51%).  Cohort 3: Male (75%). | The image reconstruction quality and diagnostic accuracy increased with the size of the latent space. With 54 parameters in the latent space, the diagnostic accuracy was 92.0 ± 2.3% with a sensitivity of 90.0 ± 2.4% (at 95% specificity), and the corresponding Dice coefficient for the reconstructed images was 0.86 ± 0.04 |
| George et al., *IEEE J Biomed Health Inform*., 2020 | An attention-guided DL model was trained using 3D-OCT volumes of ONH | Glaucoma vs. nonglaucoma | Diagnosis of glaucoma vs. healthy | 3,782 OCT volumes are split into a training, validation and testing subsets, containing 3031 (healthy: 325, POAG: 2706), 379 (healthy: 47, POAG: 332) and 372 (healthy: 55, POAG: 317) scans, respectively | Not reported | The glaucoma detection model achieved an area under the curve (AUC) of 93.8% compared with 86.8% for a baseline model without the attention-guided component |
| Mariottoni et al., *Sci Rep.*, 2021 | A ResNet50 CNN was trained using SDOCT of RNFL and SAP pairings | Predicted glaucoma vs. suspect vs. normal | Two fellowship-trained glaucoma specialists graded SDOCT and SAP pairs as normal, suspect, or glaucoma based on their clinical judgement | The training/validation sample (80%) had 7712 fundus photos/ 2342 eyes/1620 subjects;  Testing sample (20%) had 2118 fundus photos/ 585 eyes/ 405 subjects. Of the 585 eyes in the test sample, 305 (52%) had GON and 280 (48%) were normal. | Training/Validation: Glaucoma:  Female (53.7%); African American (31.3%), Caucasian (56.7%), Other race (12%);  Normal:  Female (64.8%); African American (30.6%), Caucasian 62.1%); Other race (7.3%).  Test:  Glaucoma:  Female (48.8%); African American (30.5%), Caucasian (56.8%), Other race (12.7%);  Normal: Female (70.3%); African American (28.6%), Caucasian (63.5%), Other race (7.8%). | The DL algorithm had an age-adjusted AUC of 0.92 (95% confidence interval: 0.88, 0.95) in the test sample. For a 95% specificity, the model had 77.3% sensitivity. The AUC values increased with worse levels of disease severity, achieving maximal age-adjusted AUC of 0.96 and sensitivity of 85.1% (at 95% specificity) among eyes with severe glaucoma. |
| García et al., *Comput Methods Programs Biomed.,*2021 | A CNN including the VGG16 architecture was trained with circumpapillary OCT in multiple stages: first a slide-level feature extractor, and then a volume-based predictive model, with Long Short-Term Memory (LSTM) networks | Predicted glaucomatous vs. healthy eyes | Circumpapillary OCT labeled as glaucomatous vs. healthy | 52 images (20 glaucomatous and 32 healthy) were grouped in an independent set to test the model. In each iteration of the training set (58 glaucomatous and 99 healthy eyes) were employed to train a specific model and 15 with glaucoma and 25 normal images to validate it in order to monitor and prevent overfitting.  Once the five iterations were attained, the entire training data set was used (197 circumpapillary samples) to train the final model with the architecture and parameters that reported the best performance during the internal cross-validation stage.  The final model was validated with the test set and evaluated with the external circ-DB-2 database, which is composed of 143 glaucomatous and 193 healthy circumpapillary OCT images | Database 1: Males (42.54%); Caucasian (100%).  Database 2:Males (43.22%); Caucasian (100%).  Database 3: Males (44.50%); Caucasian (100%) | The feature extractor reports AUC values higher than 0.93 both in the primary and external test sets. Otherwise, the proposed end-to-end system based on a combination of CNN and LSTM networks achieves an AUC of 0.8847 in the prediction stage, which outperforms other state-of-the-art approaches intended for glaucoma detection |
| Raja et al., *IEEE Trans Biomed Eng.,*2021 | A Hybrid CNN RAG-Netv2was trained using ONH SD-OCT scans | The Hybrid CNN to extracted RNFL, GCIPL and GCC regions, and classified against glaucoma; then SVM graded the scans into glaucoma severity (healthy vs. early vs. advanced glaucoma) using the atrophy in the RNFL, GCIPL and GCC profiles | Clinically verified healthy vs. glaucomatous SD-OCT with the markings and annotations of four expert ophthalmologists | 196 images / 169 eyes (50 healthy and 146 glaucoma) / 101 subjects.  Training (70%) and Testing (30%) | Not reported | The proposed framework achieved an F1 score of 0.9577 for diagnosing glaucoma, a mean dice coefficient score of 0.8697 for extracting the RGC regions, and an accuracy of 0.9117 for grading glaucomatous progression. Furthermore, the performance of the proposed framework is clinically verified with the markings of four expert ophthalmologists, achieving a statistically significant Pearson correlation coefficient of 0.9236. |
| Olivas et al., *Int Ophthalmol.,* 2021 | Inception V3 and MobileNet CNNs were further trained using RNFL thickness maps from OCT images | Predicted glaucomatous vs non-glaucomatous | OCT RNFL thickness maps were classified as glaucoma vs. nonglaucoma by an expert | 260 images / 260 eyes used for training, 52% of right-eye images have moderate-stage glaucoma and 48% have severe-stage glaucoma, and 60% of left-eye images have moderate-stage glaucoma and 40% have severe-stage glaucoma.  In the case of the images used for prediction, 68% of right-eye images have moderate-stage glaucoma and 32% have severe-stage glaucoma, and 65% of left-eye images have moderate-stage glaucoma and 35% have severe-stage glaucoma.  50 images per class were used for training.  Fifteen images per class, different than the ones used in the training stage, were used for running predictions | All participants were Mexican | The evaluation results of the MobileNet model for the left eye are, accuracy: 86%, precision: 87%, recall: 87%, and F1 score: 87%. MobileNet model applied to the right eye showed an accuracy of 90%, precision of 90%, recall of 90%, and F1 score of 90%. The evaluation results of the Inception V3 model for the left eye are, accuracy: 90%, precision: 90%, recall: 90%, and F1 score: 90%. The evaluation results of the Inception V3 model for the right eye are, accuracy: 90%, precision: 90%, recall: 90%, and F1 score: 90% |
| Thakoor et al., *Transl Vis Sci Technol*., 2021 | CNNs previously trained using OCT retinal nerve fiber layer (RNFL) probability maps and newly developed CNNs trained on OCT circumpapillary B-scan images were applied to an external test set | Predicted glaucomatous vs. nonglaucomatous | Expert(s) viewed a 3D Wide Glaucoma Report with visual field test points and rated eyes on a scale from 0-100% where “non-glaucomatous” was <50% and “glaucomatous” was >50% | OCT B-scan dataset: 771 scans / 771 eyes.  OCT RNFL Map dataset: 737 eyes.  Training (60%), Validating (20%), and Testing (20%) | Not reported | Training with data augmentation and training on confidently rated images enhanced the accuracy of the CNNs for glaucoma detection on a new dataset by 5% to 9%. CNN performance was optimal when a similar RS was used to establish labels both for the training and the testing sets. However, interestingly, the CNNs described here were robust to variation in the RS |
| Gaddipati et al.,  *Annu Int Conf IEEE Eng Med Biol Soc*., 2019 | A Capsule Network was trained to process 3-D Volume OCT scans of the optic nerve head – both the whole OCT volume and a resized volume; Performance was compared to a D3ResNet-50 and 3D CNN | Predicted glaucoma vs. healthy control | Clinical diagnosis of glaucoma vs. healthy controls | Total dataset: 253 volumes – 164 healthy, 89 glaucoma;  Optovue OCT: 128 volumes of ONH (95 healthy, 33 glaucoma); Spectralis Heidelberg; 125 volumes of ONH (69 healthy, 56 glaucoma).  Training - 165 volumes (106 healthy; 59 glaucoma);  Validation - 38 volumes (26 healthy, 12 glaucoma); Testing – 50 volumes (32 healthy, 18 glaucoma) | Not reported | The Capsule Network (Proposed) operating on the whole volume scan performed better than the Capsule network (Resized input) operating on the smaller resized image but training time was longer; both outperformed the 3D CNN and 3D ResNet-50. Capsule Network (Proposed) had AUC 0.973, Acc 0.94, Sensitivity 0.89, and Specificity 0.96 for diagnosing glaucoma on 3D volume OCTs of the ONH. |
| García et al., *Artif Intell Med.,* 2021 | OCT-based hybrid network combining hand-driven and deep learning algorithms was trained on the circumpapillary OCT B-scans of the RNFL; performance was compared to Fine-tuned VGG16 and RAGNet as well as machine learning program trained with RNFL | Predicted Glaucoma Severity – Advanced Glaucoma vs. Early Glaucoma vs. Healthy | Two senior ophthalmologists annotated the circumpapillary OCT B-scans for glaucoma severity in clinically diagnosed primary open angle glaucoma and healthy subjects | Total – 58 healthy, 52 early, 46 advanced;  Database 1 – 32 healthy, 28 early, 25 advanced;  Database 2 – 26 healthy, 24 early, 21 advanced | Total – Male (46.15%);  Database 1 – Male (54.12%);  Database 2 – Male (36.6%). | Compared to the other networks, the OCT-hybrid network had a similar or often greater Accuracy/ Sensitivity/ specificity for detection of healthy (0.9459 / 1 / 0.9091), Early glaucoma (0.8919/0.7500/0.9600), and Advanced glaucoma (0.9459/0.900/0.9630). |
| **STRUCTURAL ANALYSIS** | | | | | | |
| **Author, *Journal*, Year** | **Input used for training deep learning algorithm** | **Deep learning output** | **Ground Truth/Reference Standard** | **Datasets for training and testing** | **Dataset demographics** | **Main Study Finding(s)** |
| Soltanian-Zadeh S, et al., *Optica,* 2021 | A weakly supervised deep learning model (WeakGCSeg) was trained using manually graded ganglion cell layer (GCL) soma locations on adaptic optics (AO) with OCT and scanning light ophthalmoscopy (SLO) | Detected GCL soma | Two graders marked the soma locations on AO-OCT | Indiana University dataset: 8 healthy controls / volumes (3 locations /volume);  US FDA dataset: 5 glaucoma patients with hemifield defect (10 volumes) and 4 healthy age-matched subjects (6 volumes) | Not reported | For both healthy and glaucoma eyes, the WeakGCSeg performance was similar to the human gradings (P=0.125 and P=0.063). However, the WeakGCSeg algorithm performed automated segmentation of individual somas and was significantly faster than manual marking of somas (speed <3 min/volume). |
| Heisler et al., *Biomedical Optics Express*, 2020 | A Semi-supervised GAN (SS Pix2Pix GAN) was trained to segment 3D OCT volume B-scans of the optic nerve head and its performance was compared to a standard U-Net and GAN. | Segmentation of the inner limiting membrane, posterior boundary of RNFL, Bruch’s membrane, Bruch’s membrane opening, and choroid-sclera boundary | Automated segmentation that was examined and manually corrected by trained graders | Peripapillary layer segmentation dataset:  Glaucoma – 26354 B-scans/69 volumes/30 subjects/16 eyes; Control – 46287 B-scans/128 volumes/25 eyes/13 subjects;  BMO segmentation dataset:  Glaucoma – 10880 radial frames / 136 volumes / 65 eyes / 34 subjects;  Control – 11600 radial frames / 145 volumes / 30 eyes / 16 subjects;  Unlabeled Dataset – Glaucoma - 6796 B-scans / 22 volumes / 22 eyes / 13 subjects;  Control – 2662 B-scans / 8 volumes / 8 eyes / 4 subjects.  Training set 1 (800 training and 200 validation B-scans); Training set 2 (8000 training and 2000 validation B-scans). Testing set (171 volumes/40 eyes/20 subjects); equal number of control and glaucoma B-scans in training sets. | Peripapillary layer segmentation dataset:  Glaucoma - Male:female (9:7); Control – Male:female (6:7);  BMO Segmentation Dataset – Glaucoma – Male:female (19:15);  Control – Male:female (8:8);  Unlabeled dataset: Glaucoma – Male:female (7:6); Control – Male:female (1:3). | The semi-supervised Pix2Pix GAN had the best Dice Coefficients for ILM-RNFL, RNFL-BM, and BM-CS (0.8958, 0.9394, 0.8440); The Pix2Pix GAN had the best dice coefficient for the start of the ILM (0.9730), and the U-Net the best Choroid-scleral boundary (0.9644). |
| Cheong et al., *TVST* 2020. | Deshadow generative adversarial network (GAN) was trained to remove blood vessel shadows in OCT B-scans of ONH | Generated OCT with less shadows based on intralayer contrast | Intralayer contrast was quantified from 0 (shadow-free) to 1 (strong shadow) in RNFL, photoreceptors, inner plexiform layer, and retinal pigment epithelium by a single grader. | 13 healthy subjects; training set – 2328 multiframe B-scans from 24 3D volumes; Test sent – 291 multiframe B-scans from three 3D volumes | Not reported | Blood vessel shadows were corrected by Deshadow GAN and this was confirmed qualitatively and quantiatively. The intralayer contrast improved 33.7 +/- 6.81%, 28.8 +/- 10.4%, 35.9 +/- 13% and 43 +/- 19.5% for RNFL, IPL, PR and RPE respectively. |
| Thompson et al., *JAMA*, 2020 | ResNet-34 was trained using SD-OCT images without segmentation lines | Predicted RNFL thickness; glaucomatous vs normal eyes | RNFL thickness parameters | 20,806 images / 1154 eyes / 635 participants.  Training (50%), validation (20%), and testing (30%) | Healthy: Women (59.8%); African-American (19.9%).  Glaucoma: Women (53.3%); African-American (24.3%) | The DL algorithm had a significantly higher area under the receiver operating characteristic curve than global RNFL thickness (0.96 vs 0.87; difference = 0.08, 95% CI: 0.04-0.12) and each RNFL thickness sector for discriminating between glaucoma and controls (all P < .001). At 95% specificity, the DL algorithm (81%; 95% CI: 64%-97%) was more sensitive than global RNFL thickness (67%; 95% CI: 58%-76%) |
| Rezapour et al., *Sci Rep*., 2021 | SALSA-deep was developed using BCDU-Net which was trained with SD-OCT RNFL circle scans | Identified the basement membrane and posterior boundary of the choroid to calculate peripapillary choroidal thickness (PCT) | Manually segmented the Bruch’s membrane and posterior boundary of the choroid on SD-OCT circle B-scans | 452 glaucoma eyes of 277 patients were included with 145 eyes (81 patients) in the no axial myopia group, 214 eyes (128 patients) in the mild axial myopia group and 93 eyes (68 patients) in the high axial myopia group | No axial myopia: Female (65.4%); European (67.9%), African (23.5%), Asian (7.4%), Other/unknown race (1.2%).  Mild axial myopia: Female (43.0%); European (68.8%), African (17.2%), Asian (12.5%), Other/unknown race (1.6%).  High axial myopia: Female (47.1%); European (66.2%), African (13.2%), Asian (17.6%), Other/unknown race (2.9%) | High myopic optic discs were more oval and had larger BMO tilt than mild and non-myopic discs (both p < 0.001). Mean peipapillary choroidal thickness (PCT) was thinnest in high myopic eyes followed by mild and non-myopic eyes (p < 0.001). BMO rotation angle, global retinal nerve fiber layer (RNFL) thickness and BMO-minimum rim width (MRW) were similar among groups. Temporal RNFL was thicker and supranasal BMO-MRW was thinner in high myopic eyes. BMO tilt and PCT showed moderate and temporal RNFL and nasal BMO-MRW showed weak but significant associations with AL in multivariable analyses (all p < 0.05). Large BMO tilt angle and thin PCT are characteristics of highly myopic discs and were not associated with severity of glaucoma |
| Schottenhamml et al., *Biomed Opt Express,* 2021 | Multiple different CNNs (DenseNet121, DenseNet161, ResNet-152, ResNext-50-32x4d, and WideResNet-101-2) were trained using *en face* 3x3 OCTA images of different retinal projections (of the whole retina, the superficial vascular plexus, the intermediate capillary plexus, and the deep capillary plexus) centered around the fovea | Extracted features for distinguishing glaucoma vs. healthy | Glaucoma vs. healthy based on prior clinical exam | 259 eyes / 199 subjects (75 eyes of 74 healthy subjects and 184 eyes of 125 glaucoma patients).  Training (60%), Validation (20%), and Testing (20%) | Female (50.75%) | On the whole retina projection and the superficial vascular plexus projection, CNNs outperform the handcrafted features presented in the literature. The AUC was 0.967 ± 0.026 for the the superficial vascular plexus projection, 0.923 ± 0.022 for retina, 0.915 ± 0.046 for intermediate  capillary plexus, and 0.910 ± 0.031 for deep capillary plexus. |
| Yow et al., *Annu Int Conf IEEE Eng Med Biol Soc.*,2020 | A U-Net based CNN was trained using circumpapillary OCT scans extracted from SS-OCT volumetric scans | Automated RNFL segmentation | Manually annotated cross-sectional RNFL scans | 130 eyes (36 glaucoma eyes and 94 non-glaucoma eyes).  All scans were used for training and validating. | Not reported | The diagnostic accuracy in detecting glaucoma using manually annotated RNFL thickness was 0.84 (95% CI: 0.74 to 0.92). For the automated RNFL segmentation, the diagnostic accuracy was 0.84 (95% CI: 0.75 to 0.91). There was no statistically significant difference (P = 0.89) found between the AUCs for the manual annotation and automated segmentation. |
| Mariottoni et al., *Sci Rep*., 2020 | ResNet34 was trained using raw SDOCT B-scans of peripapillary RNFL without the segmentation lines | Predicted RNFL thickness | Global RNFL thickness on SDOCT | The training/ validation dataset: 13,262 images/476 subjects/897 eyes, split at the patient level for training (80% of the sample) and validation (20%). Performance of the algorithm was evaluated in images from 441 eyes of 230 subjects, distributed between three different Test Sets. The Test Set 1 contained 11,010 images Test Set 2 had 237 images with segmentation errors and Test Set 3 had 776 low-quality images | Training: Female (56.03%); African-American (18.4%).  Validation: Female (60.64%).;African-American (17.71%).  Testing: Female (55.65%). African-American (27.39%) | The algorithm was tested in three different sets: (1) images without segmentation errors or artifacts, (2) low-quality images with segmentation errors, and (3) images with other artifacts. In test set 1, segmentation-free RNFL predictions were highly correlated with conventional RNFL thickness (r = 0.983, P < 0.001). In test set 2, segmentation-free predictions had higher correlation with the best available estimate (tests with good quality taken in the same date) compared to those from the conventional algorithm (r = 0.972 vs. r = 0.829, respectively; P < 0.001). Segmentation-free predictions were also better in test set 3 (r = 0.940 vs. r = 0.640, P < 0.001) |
| Jammal et al., *Sci Rep.*, 2019 | A ResNet34 was trained using SDOCT B-scans of the segmented peripapillary RNFL | Determined the probability of a segmentation error in RNFL and highlighted the location of artifacts with heatmaps | Human graders labeling SD-OCT B-scans for RNFL segmentation errors | 25,250 B-scans. Training (50%) and Testing (50%) | Normal: Female (54.4%); Caucasian (71.3%), African-American (28.7%).  Suspect: Female (58.0%); Caucasian (68.1%), African-American (31.9%).  Glaucoma: Female (49.4%); Caucasian (56.0%), African-American (44.0%).  Overall: Female (54.7%); Caucasian (65.5%), African-American (34.5%) | The DL algorithm had an area under the ROC curve of 0.979 (95% CI: 0.974 to 0.984) and an overall accuracy of 92.4%. |
| Devalla et al., *IOVS*, 2018 | A 2-dimensional CNN was trained using horizontal B-scan OCT images of the optic nerve head (ONH) | Automatically and simultaneously stained six important neural and connective tissue structures in OCT images of the ONH | Manual segmentation of ONH layers | 40 healthy eyes / 40 patients. 60 glaucoma eyes / 60 patients.  Training (40%) and Testing (60%) | Not reported | For all tissues, the dice coefficient, sensitivity, specificity, IU, and accuracy (mean) were 0.84 ± 0.03, 0.92 ± 0.03, 0.99 ± 0.00, 0.89 ± 0.03, and 0.94 ± 0.02, respectively. The algorithm performed significantly better when compensated images were used for training (P < 0.001) |
| Sedai et al., *Ophthalmol Glaucoma*, 2020 | Four different forecasting models were trained using clinical (age and intraocular pressure), structural (cpRNFL thickness derived from scans as well as deep learning-derived OCT image features), and functional (visual field test parameters) data and the intervisit interval in a longitudinal cohort | Predicted future cpRNFL thickness in healthy, glaucoma suspect, and glaucoma participants | True average thickness of cpRNFL | 1089 participants (651 glaucoma patients, 404 glaucoma suspects, and 34 healthy controls).  Training (78.87%) and Testing (21.13%) | Healthy: Female (58.54%).  Glaucoma suspect: Female (56.30%).  Glaucoma: Female (53.65%) | The best forecasting model of cpRNFL was obtained using 3 visits and incorporated deep learning-derived OCT image features. The mean error was 1.10 ± 0.60 μm, 1.79 ± 1.73 μm, and 1.87 ± 1.85 μm in eyes of healthy, glaucoma suspect, and glaucoma participants, respectively. This method significantly outperformed the linear trend-based estimation model for glaucoma suspect and glaucoma participant (P < 0.001), which showed a mean error of 1.55 ± 1.16 μm, 2.4 ± 2.67 μm, and 3.02 ± 3.06 μm in the 3 groups, respectively. The Pearson’s correlation coefficient between the forecasted value and the actual measured thickness of RNFL was ρ = 0.96 (P < 0.01), ρ = 0.95 (P < 0.01), and ρ = 0.96 (P < 0.01) for the 3 groups, respectively. |
| Devalla et al., *Biomed Opt Express*, 2018 | A deep learning custom U-NET (DRUNET) algorithm was trained using SD-OCT horizontal B-scans through the ONH | Automatically and simultaneously stained six important neural and connective tissue structures on OCT images of the ONH | Manual segmentation of the horizontal B-scan of the ONH | 40 normal controls, 41 subjects with POAG, and 19 subjects with PACG. | Not reported | The overall Dice coefficient (mean of all tissues) for the DRUNET algorithm was 0.91 ± 0.05 when assessed against manual segmentations performed by an expert observer |
| Devalla et al., *Biomed Opt Express*, 2020 | Designed an “enhancer” DL based image enhancement network to enhance OCT image quality and harmonize image characteristics across OCT B-scans of ONH from three devices (using Spectralis, Cirrus, and RTVue);  -Then developed ONH-Net, in which a 3D U-Net was trained using SD-OCT volumes of the ONH to segment 6 ONH tissues | Automatically segmented SD-OCT volumes of the optic nerve head into 6 ONH tissues | Manual segmentation of SD-OCT volumes into 6 ONH tissues | 450 subjects/eyes/ SD-OCT volumes (225 healthy, 225 glaucoma).  Training and Testing of the image enhancement network – 390 (86.7%).  Training and testing (the 3D segmentation framework – 60 13.3%). | Not reported | When the ‘enhancer’ was used to preprocess the OCT images, the ‘ONH-Net’ trained on any of the 3 devices successfully segmented ONH tissues from the other two unseen devices with high performance (Dice coefficients > 0.92) |
| Lazaridis, et al., *Med Image Anal*. 2021 | An ensemble of cyclical generative adversarial networks with or without a cycle-consistent perceptual loss were trained on Time-Domain OCT (TDOCT) B-scans of RNFL which were converted to synthesized SDOCT. | Predicted RNFL thickness on the synthesized OCT B-scan | RAPID Test-retest dataset – glaucoma cohort (diagnosis based on VF testing, imaging and clinical assessment) with RNFL thickness from acquired Stratus TDOCT;  United Kingdom Glaucoma Treatment Study (UKGTS) – new diagnoses of glaucoma, monitored with visual field, OCT of ONH, optic disc photography, and tonometry and randomized to treatment with latanoprost vs. placebo. | RAPID test-retest dataset –77 open angle glaucoma patients, 148 eyes, 4902 TDOCT and 1789 SDOCT scans.  UKGTS dataset – 373 open angle glaucoma patients, 78,415 TDOCT | Not reported | In the RAPID test-retest dataset, the proposed ensemble GAN+Perceptual method had a significantly better performance with less test-retest variability in RNFL predictions (Limits of Agreement (6.57-7.59), Mean Difference 0.39, Mean Standard Deviation 1.06) relative to the original TDOCT images or other explored methods such as GAN, Wasserstein GAN, and Wasserstein GAN+Perceptual. In the UKGTS dataset, the ensemble method predicted a significant difference in mean rate of change of RNFL (0.282, 95% CI: 0.0003, 0.5654), p<0.05) between latanoprost treatment and placebo group on the synthesized SDOCT; this difference was not significant using the original RNFL measurements on TDOCT (0.107, 95% CI: -0.358, 0.574). Thus, the proposed method enabled detection of faster rates of RNFL thinning in the placebo group relative to latanoprost group which the original TDOCT measurements failed to detect. |
| Lazaridis et al., *Ophthalmol Glaucoma*. 2021 | An ensemble of cyclical generative adversarial networks (GANs) was trained on TDOCT-SDOCT pairs in the training dataset and applied to TDOCT images converted to synthesized SDOCT in the test dataset. | Predicted RNFL thickness on the synthesized OCT B-scan | RAPID Test-retest dataset – glaucoma cohort (diagnosis based on VF testing, imaging and clinical assessment) with RNFL thickness from acquired Stratus TDOCT;  United Kingdom Glaucoma Treatment Study (UKGTS) – new diagnoses of glaucoma, monitored with visual field, OCT of ONH, optic disc photography, and tonometry and randomized to treatment with latanoprost vs. placebo. | RAPID test-retest– Training/Validation dataset: 77 glaucoma patients, 148 eyes, 4645 TDOCT and 1721 SDOCT;  UKPDS, Test Dataset– 284 newly diagnosed open angle glaucoma patients – 141 latanoprost treatment and 143 placebo | Not reported | Image enhancement improved the agreement of predicted RNFL from TDOCT which was converted to a synthesized SDOCT with the original SDOCT RNFL measurements. The 95% limits of agreement between  synthesized SDOCT and SDOCT were (8.11, -6.73), and between the original TDOCT and SDOCT were (26.64, -22.95). The mean difference in the rate of RNFL change between UKGTS treatment and placebo arms with synthesized SDOCT was 0.43 (p=0.0017) and with TDOCT was 0.24 (p=0.11). |
| Mao et al. *Biomed Opt Express*, 2019 | A U-net deep learning-based noise reduction algorithm in combination with a vessel shadow compensation method and 3-D segmentation technique was created to automatically segment the lamina cribrosa in enhanced volume OCT B-scans of the optic nerve head. | Segmented the anterior surface of the lamina cribrosa on 3-D optic nerve head OCT volume B-scans | OCT images with automatic lamina cribrosa segmentation were reviewed by glaucoma specialists and scored as good, bad or uncertain. The ground truth was defined as the automatic segmentation boundaries after manual correction by the grading experts. | 36 subjects /72 eyes: 33 eyes with normal tension glaucoma, 25 eyes with primary open angle glaucoma, 6 eyes with exfoliation glaucoma, and 5 glaucoma suspects. | 22 (61.1%) Female | After combining the noise reduction algorithm, the shadow compensation, and contrast enhancement technique, expert graders were able to identify the anterior border of the lamina cribrosa in 98.3% of enhanced B-scans which was better than the raw B-scans (87.2%). A 90.6% accuracy was achieved in a validation study of 180 B-scans from 36 subjects, which was significantly better than the 64.4% accuracy achieved in the raw B-scan images. |
| Zhang, et al. *IEEE J Biomed Health Inform.* 2020. | A biomarker-infused global-to-local network (Bio-Net) for the choroid segmentation was trained to segment the choroid on SD-OCT retinal/choroid images. In addition, a deep learning pipeline was trained to locate shadows then predict the choroidal vasculature with an edge-to-texture generative adversarial inpainting network. | Automatic segmentation of the choroid; shadow localization and removal | Swept source OCT volume scan of the 6x6 mm^2^ fovea was manually segmented by expert graders into the retinal and choroidal layers as well as the shadows on the RPE projection images | 34 healthy subjects/68 eyes/136 OCT volume scans of the fovea - OCT imaging was acquired in an upright position (normal intraocular pressure) and an upside-down position (high intraocular pressure). | Not reported | The dice index (92.74 ± 0.02) and intersection-over-union (86.47 ± 0.04) were higher for Bio-Net indicating better performance than other published methods. Also, the average-unsigned-surface-detection-error (AUSDE) was lowest for the choroidal-scleral interface (4.31 ± 0.02) and Bruch’s membrane (0.77± 0.02) indicating less pixel-wise mismatch between the segmented choroid boundary and ground truth. |
