# Supplementary material for: Deep learning and optical coherence tomography in glaucoma: Bridging the diagnostic gap on structural imaging
Source: Front Ophthalmol (Lausanne). 2022 Sep 21;2:937205. doi: 10.3389/fopht.2022.937205 (PMC11182271; doi:10.3389/fopht.2022.937205)
Supplement: Supplementary file 2 [file Table_2.docx]

**Table 2: Deep learning algorithms trained to grade angle morphology or identify angle anatomy on anterior segment or swept source OCT of the anterior chamber angle**

| **OPEN VS. CLOSED** | | | | | | |
| --- | --- | --- | --- | --- | --- | --- |
| Author, *Journal*, Year | Input used for training deep learning algorithm | Deep learning output | Ground Truth/Reference Standard | Datasets for training and testing | Dataset demographics | Main Study Finding(s) |
| Li et al., *Transl Vis Sci Technol.,* 2021 | Inception V3 CNN was trained with horizontal scans from AS-OCT images; additional networks were also trained for comparison (VGG16, ResNet-18, ResNet-50) | Predicted open angle vs. narrow angle vs. angle-closure | The angles were classified as open, narrow, or angle-closure on gonioscopy by two specialists | 6,000 images / 3,000 eyes / 1,826 subjects. Training (80%) and testing (20%) | Female (76.8%) | The Inception V3 algorithm reached a sensitivity of 0.999 and specificity of 1.000 for angle closure detection. The overall classification of the proposed method in open angle, narrow angle, and angle-closure classifications reached a sensitivity of 0.989 and specificity of 0.995. Additionally, the sensitivity and specificity reached 1.000 and 1.000 for angle-closure, 0.983 and 0.993 for narrow angle, and 0.985 and 0.991 for open angle. |
| Porporato et al., *Br J Ophthalmol*., 2021 | VGG-16 network was trained using A-scans from SS-OCT images | Predicted open vs. closed | Gonioscopy grade of open vs. closed | 39,936 images / 128 subjects | Female (57.4%).  Asian (82.1%) | The AUC of the deep learning algorithm was 0.85 (95% CI: 0.80 to 0.90), with sensitivity of 83% and a specificity of 87% to classify gonioscopic angle closure with the optimal cut-off value of >35% of circumferential angle closure |
| Randhawa et al., *Br J Ophthalmol.,* 2021 | A multiclass CNN based on ResNet-18 was trained using AS-OCT images | Predicted open vs. closed angle | Gonioscopy grades of open vs. closed | Three datasets: 640 images (311 open and 329 closed) / 127 subjects; 10,165 images (9595 open and 570 closed) / 1,318 subjects; 300 images (234 open and 66 closed) / 40 subjects | CHES training:  Female (68.5%); Asian (100%).  CHES Testing:  Female (66.1%); Asian (100%).  Singapore:  Female (63.3%); Asian (87.6%), Indian (7.1%), Malay (2.7%).  USC: Female (55.0%); Asian (32.5%), Non-Hispanic white (22.5%), Black (12.5%), Hispanic (32.5%) | The classifier achieved similar performance in the CHES (AUC=0.917), Singapore (AUC=0.894) and USC (AUC=0.922) cohorts. Standardizing the distribution of gonioscopy grades across cohorts produced similar AUC metrics (range 0.890–0.932). The agreement between the CNN classifier and two human examiners (Ҡ=0.700 and 0.704) approximated inter-examiner agreement (Ҡ=0.693) in the USC cohort |
| Xu et al., *Am J Ophthalmol.,* 2019 | Three competing convolutional neural networks were trained using AS-OCT images: ResNet-18, a custom 14-layer CNN, and an Inception-v3 model plus logistic regression combination algorithm | Predicted Schaffer grades 0-4 and binary probabilities of open vs. closed angles | Open (Shaffer grade 0-1) vs. closed (Shaffer 2-4) angle grades on gonioscopy by expert graders | 4,036 images (1,943 open, 2,093 closed). Prior to classifier training, 85% of images were segregated into a cross-validation dataset. The remaining 15% of images were segregated into an independent test dataset.  80% and 20% of the cross-validation dataset were used for training and validation. | Female (68.3%); Chinese-American (100%) | For detecting gonioscopic angle closure, the ResNet-18 classifier had the best performance, and achieved an AUC of 0.933 on the cross-validation dataset and 0.928 on the test dataset. For detecting PACD based on two- and three-quadrant definitions, the ResNet-18 classifier achieved AUCs of 0.964 and 0.952, respectively, on the test dataset |
| Shen et al., *Br J Ophthalmol.,* 2021 | Previously described deep learning classifier (Xu et al., *Am J Ophthalmol.,*  2019) that was trained using AS-OCT images | Predicted open vs. closed angles | Open (Shaffer grade 0-1) vs. closed (Shaffer 2-4) angle grades on gonioscopy | 584 images / 127 subjects | Female (65.2%) | This is an analysis of the biometric differences between images that were correctly and incorrectly classified with closed angles by a previously described deep learning algorithm. False Positives (FP) resembled True Positives (TP) more than False Negatives (FN) and True Negatives (TN) in terms of anterior segment parameters (steeper Iris Curvature and higher Lens Vault), but resembled TN more than TP and FN in terms of angle parameters (wider Angle Opening Distance). Models for detecting FP (AUC=0.752) and FN (AUC=0.838) improved classifier accuracy from 84.8% to 89.0% |
| Fu et al., *Am J Ophthalmol.,* 2019 | VGG-16 network was trained using AS-OCT images | Predicted angle closure vs. open angle on AS-OCT | Single observer determined label of open-angle or angle-closure on AS-OCT | 4,135 images (each with 2 ACA regions) / 4,226 eyes / 2,113 subjects (8270 anterior chamber angle images with 7375 open-angle and 895 angle-closure) | Female (54.9%); Chinese, Indian, and Malay (100%) | The AUC of the system using quantitative features was 0.90 (95% CI: 0.891–0.914) with a sensitivity of 0.79 ± 0.037 and a specificity of 0.87 ± 0.009, while the area under the receiver operating characteristic curve of the deep learning system was 0.96 (95% CI: 0.953–0.968) with a sensitivity of 0.90 ± 0.02 and a specificity of 0.92 ± 0.008, against clinicians' grading of AS-OCT images as the reference standard |
| Fu et al., *IEEE TransCybern.,* 2020 | A multilevel deep network (MLDN) was trained using AS-OCT images. Three parallel subnetworks extracted hierarchical representations from different clinically relevant regions in an AS-OCT image, including the global anterior segment structure, local iris region, and anterior chamber angle patch. The output maps were concatenated as input into one fully connected final layer to provide a prediction of angle-closure. | Open vs. closed angle | AS-OCT images were labeled as open-angle or angle-closure by expert graders | Two datasets: 4135 images / 2113 subjects. (7375 open-angle and 895 angle-closure ACA images). 701 images / 202 subjects (1102 open-angle and 300 angle-closure ACA images). Each dataset was divided randomly into training and testing sets at the patient level | Not reported | The MLDN showed an AUC of 0.9619 (95% CI: 0.9499–0.9711) on the Visante AS-OCT dataset and 0.9524 (95% CI: 0.9208–0.9718) on the Cirrus HD-OCT dataset, respectively. |
|  | | | | | | |
| **OPEN VS. CLOSED – APPOSITIONAL VS. SYNECHIAL ANGLE CLOSURE** | | | | | | |
| Author, *Journal*, Year | Input used for training deep learning algorithm | Deep learning output | Ground Truth/Reference Standard | Datasets for training and testing | Dataset demographics | Main Study Finding(s) |
| Hao et al., *Med Image Anal*., 2021 | Multisequence deep network (MSDN), which consisted of two subnetworks (CNN encoder and Convolutional Long Short Term Memory i.e. ConvLSTM), was trained using AS-OCT images | Predicted open vs. appositional vs. closed/synechial | Graded ACAs into open, appositionally narrow, and synechial angles on AS-OCT | 66 eyes: 21 eyes (POAG) were open, 13 eyes (PACS) were appositional, and 32 eyes (PAC/PACG) were closed) / 60 subjects | Not reported | The accuracy of the multi-sequence deep network for grading anterior chamber angle into open, appositional, and synechial was 91.46% in the dark dataset, 91.08% in the bright dataset, and 92.13% in the combined dataset |
| Yang et al., *Front Med (Laussane*), 2021 | Two deep learning classifiers were trained using SS-OCT images of the anterior chamber angle | First DL classifier detected static angle closure vs. open angle; Second DL classifier distinguished appositional from synechial angle closure | Gonioscopy grading of open vs. closed – appositional vs. synechial | 77613 images / 439 eyes/278 patients which contained 165 normal, 99 primary angle closure suspect, 85 primary angle closure, and 90 primary angle closure glaucoma eyes; First DL classifier – 70% training, 15% validation, 15% test; Second DL classifier – 80% training 10% validation, 10% test | Normal - Male (46.3%),  PACS - Male (26.8%),  PAC - Male (33.8%),  PACG – Male (34.9%).  Chinese (100%) | First DL classifier had an AUC 0.963 (95% CI: 0.954-0.972) for detection of static angle closure; The second DL classifier had an AUC 0.873 (95% CI: 0.864-0.882) for distinguishing appositional from synechial closure |
| Li et al., *Ophthalmol*, 2022 | A 3-D deep learning based automated digital gonioscopy system (DGS) was trained using AS-OCT images of anterior chamber | Task 1: Detect static open vs. narrow angle  Task 2: Detect Non-PAS vs. PAS angle closure | Gonioscopy grading by 2 graders for Task 1 (static – narrow vs. open angle) and Task 2 (dynamic – peripheral anterior synechiae vs. appositional narrowing) | Total 1.112 million images of 8694 volume scans/2294 patients. Task 1 (4515 training, 1101 internal validation, 2222 external test) vs. Task 2 (378 training, 376 internal validation, 102 external test).    Task 1: 7838 volume scans – 4515 training (3012 open, 1503 narrow), 1101 validation (706 open, 517 close), 2222 test (1705 open, 517 close).  Task 2: 856 volume scans— 4536 training (480 PAS, 4056 non-PAS), 4512 validation (576 PAS, 3936 non-PAS), 1224 test (240 PAS, 984 non-PAS) | Race: Task 1: Open angle group – 90.8% Chinese, 2.1% Malaysian, 4.7% Indian, 2.5% Other; Narrow angle group – 94.4% Chinese, 1.8% Malaysian, 3.1% Indian, 0.7% Others.  Task 2: PAS group – 80% Chinese, 20% Thai; Non-PAS group –86.9% Chinese, 13.1% Thai.  Sex (M/F): Task 1 – Open angle 629/733; Narrow angle 172/388; Task II 21/29; Non-PAS 150/164. | Task 1: AUC 0.943, 95% CI: 0.933-0.953;  Task 2: AUC 0.902, 95% CI: 0.818-0.985 |
|  | | | | | | |
| **SCLERAL SPUR AND PLATEAU IRIS** | | | | | | |
| Author, *Journal*, Year | Input used for training deep learning algorithm | Deep learning output | Ground Truth/Reference Standard | Datasets for training and testing | Dataset demographics | Main Study Finding(s) |
| Pham et al., *Br J Ophthalmol.,* 2021 | A Full-resolution residual U-Net (FRRUnet) hybrid deep CNN trained using AS-OCT images | FRRUnet was used for detection of scleral spur while an ensemble of U-Net, FRRnet and FRRUnet was used for segmentation of the anterior segment structures (iris, corneoscleral shell, and anterior chamber) | Three human observers - trained non-expert, trained medical student, and fellowship-trained glaucoma expert - localized scleral spur on AS-OCT images | 820 images / 175 eyes / 100 patients.  Training (75.6%) and Testing (24.4%) | Male (31.91%);  Chinese (77.86%), Malay (11.42%), Indian (7.86%), Other race (2.86%) | The deep CNN was able to detect the scleral spur on unseen ASOCT images as accurately as an experienced ophthalmologist on the given test dataset and simultaneously isolated the anterior segment structures with a Dice coefficient of 95.7% |
| Wanichwecharun-gruang et al., *Transl Vis Sci Technol*.,  2021 | ResNet was trained using pairs of AS-OCT scans and ultrasound biomicroscopy | Predicted presence or absence of plateau iris on AS-OCT images | Senior glaucoma specialist labeled images of the quadrants with plateau iris on AS-OCT | 716 images / 179 eyes / 142 patients with primary angle closure disease which had remaining apposition after iridotomy. Training (77.7%) and testing (22.3%) | Female (76.8%); Asian (100%) | AUC for plateau iris was 0.95 (95% CI: 0.91, 0.99), sensitivity was 87.9%, and specificity was 97.6% |
| Xu et al., *Transl Vis Sci Technol.,*2020 | A deep CNN was trained using AS-OCT images | Predicted the Cartesian coordinates of the scleral spur on the AS-OCT images | Two graders marked the scleral spur on AS-OCT images | 18,664 images / 2333 eyes / 2096 participants.  Training (95%) and Testing (5%) | Female (65.1%) | The mean absolute prediction errors of the CNN model were 49.27 ± 42.07 µm for X-coordinates and 47.73 ± 39.70 µm for Y-coordinates. The mean absolute intra-grader variability was 52.31 ± 47.75 µm for X-coordinates and 45.88 ± 45.06 µm for Y-coordinates. Distributions of prediction errors for the CNN and intra-grader variability for the reference grader were similar for X-coordinates (P = 0.609) and Y-coordinates (P = 0.378). The mean absolute prediction error of the CNN was 73.08 ± 52.06 µm and the mean absolute intra-grader variability was 73.92 ± 60.72 µm |
