# Supplementary material for: Deep learning and optical coherence tomography in glaucoma: Bridging the diagnostic gap on structural imaging
Source: Front Ophthalmol (Lausanne). 2022 Sep 21;2:937205. doi: 10.3389/fopht.2022.937205 (PMC11182271; doi:10.3389/fopht.2022.937205)
Supplement: Supplementary file 3 [file Table_3.docx]

**Table 3: Deep learning algorithms trained with OCT data to interpret color fundus or red-free photos for glaucomatous damage**

| **RNFL Thickness** | | | | | | |
| --- | --- | --- | --- | --- | --- | --- |
| Author, *Journal*, Year | Input used for training deep learning algorithm | Deep learning output | Ground Truth/Reference Standard | Datasets for training and testing | Dataset demographics | Main Study Finding(s) |
| Lee et al., *Am J Ophthalmol.,* 2021 | The M2M algorithm was a ResNet 34 CNN that was previously trained using pairs of fundus photographs and RNFL thickness values from SD-OCT scans | In this analysis the M2M Ppredicted baseline and longitudinal RNFL thickness when evaluating a color fundus photograph from a longitudinal dataset; additional analyses considered whether glaucoma suspects converted to having glaucoma with a visual field defect. | RNFL thickness from SDOCT was the ground truth for training the model | Training set - 26,509 pairs of fundus photos / 2,521 eyes / 1,391 patients.  Testing set – Converters (196 eyes), Non-converters (876 eyes) | Converters: Female (62%); White (56%), African American (40%).  Nonconverters: Female (37%); White (61%), African American (36%). | Baseline RNFL and rate of change of predicted RNFL thickness were significantly predictive of conversion to glaucoma, with hazard ratios in the multivariable model of 1.56 per 10 μm lower at baseline (95% CI: 1.33-1.82; P < 0.001) and 1.99 per 1 μm/yr faster loss in thickness during follow-up (95% CI, 1.36-2.93; P < 0.001) |
| Medeiros et al., *Ophthalmology*, 2021 | A machine-to-machine (M2M) algorithm was developed by training ResNet50 with pairs of fundus photographs and RNFL thickness from SD-OCT scans | Predicted RNFL thickness on fundus photographs in an independent longitudinal sample in order to detect glaucoma progression | SD-OCT RNFL thickness measurements | 33,466 pairs of fundus photos /1147 eyes / 717 patients.  Training plus validation (50%), and Testing (50%) | Glaucoma suspect: Female (65.6%); White (59.5%), Black (40.5%).  Glaucoma: Female (58.2%); White (56.5%), Black (43.5%) | RNFL predictions showed an ROC curve area of 0.86 (95% CI: 0.83–0.88) to discriminate progressors from non-progressors. For detecting fast progressors (slope faster than 2 μm/year), the ROC curve area was 0.96 (95% CI: 0.94–0.98), with a sensitivity of 97% for 80% specificity and 85% for 90% specificity. For photographs obtained at the same visit, the intraclass correlation coefficient was 0.946 (95% CI: 0.940–0.952), with a coefficient of variation of 3.2% (95% CI: 3.1%–3.3%) |
| Jammal et al., *Am J Ophthalmol.,* 2020 | The M2M algorithm was previously trained using fundus photographs and SD-OCT scans | Predicted RNFL thickness; this prediction was applied to discriminate between eyes with perimetric glaucoma (reproducible visual field loss) vs. normal fields | SD-OCT RNFL thickness measurements were reference for training of M2M | 32,820 pairs of fundus photos / 2,312 eyes / 1,198 subjects. Training plus validation (80%) and testing (20%) | Female (52.7%);  Caucasian (58.6%), African American (41.4%) | The overall AUC for the M2M DL-predicted RNFL thickness was similar to that of the probability of GON given by human graders [AUC = 0.801 (95% CI: 0.757, 0.845) versus 0.775 (95% CI: 0.728, 0.823), respectively; P = 0.222], and both of them performed significantly better than the vertical C/D [AUC = 0.732 (95% CI: 0.680, 0.784)] or horizontal C/D ratio [AUC = 0.683 (95% CI: 0.628, 0.739); all comparisons P < 0.05] |
| Medeiros et al., *Ophthalmology,* 2019 | A machine-to-machine (M2M) algorithm was developed by training ResNet34 using pairs of optic disc photographs and the SD-OCT average RNFL thickness values | Predicted SDOCT average RNFL thickness to quantify glaucomatous structural damage | Actual SDOCT average RNFL thickness | 32,820 pairs of optic disc photos / 2,312 eyes / 1,198 subjects.  Training plus validation (80%) and Testing (20%) | Normal: Female (64.7%). Caucasian (56.7%). African-American (43.3%).  Glaucoma suspect: Female (60.5%). Caucasian (61.8%). African-American (38.2%).  Glaucoma:Female (53.1%). Caucasian (60.2%). African-American (39.8%) | There was a high correlation between predicted and observed RNFL thickness values (Pearson’s r=0.832; p<0.001) with a mean absolute error of the predictions of 7.39 microns. The areas under the ROC curves for discriminating glaucomatous from healthy eyes with the deep learning predictions and actual SDOCT average RNFL thickness measurements were 0.944 (95% CI: 0.912– 0.966) and 0.940 (95% CI: 0.902 – 0.966), respectively (P = 0.724). |
|  | | | | | | |
| **BMO-MRW Thickness** | | | | | | |
| Author, *Journal*, Year | Input used for training deep learning algorithm | Deep learning output | Ground Truth/Reference Standard | Datasets for training and testing | Dataset demographics | Main Study Finding(s) |
| Thompson et al., *Am J Ophthalmol.,* 2019 | A ResNet34 CNN was trained using pairs of fundus photographs with the BMO-MRW measurement from SD-OCT | Predicted the BMO-MRW thickness | The BMO-MRW global and sector thickness values from SDOCT | 9,282 pairs of optic disc photos / 927 eyes / 490 subjects.  Training plus validation (80%) and Testing (20%) | Normal: Female (67.8%); Caucasian (58.1%), African-American (41.9%).  Glaucoma suspect: Female (58.2%); Caucasian (70.8%), African-American (29.2%).  Glaucoma: Female (52.0%); Caucasian (69.6%), African-American (30.4%). | The AUCs for discriminating glaucomatous from healthy eyes with the DL predictions and actual SDOCT global BMO-MRW measurements were 0.945 (95% CI: 0.874–0.980) and 0.933 (95% CI: 0.856–0.975), respectively (P=0.587) |
|  | | | | | | |
| **mGCIPL Thickness** | | | | | | |
| Author, *Journal*, Year | Input used for training deep learning algorithm | Deep learning output | Ground Truth/Reference Standard | Datasets for training and testing | Dataset demographics | Main Study Finding(s) |
| Lee et al., *Sci Rep.,* 2020 | A Hybrid deep learning model (HDLM) developed – an Inception-ResNet-v2 CNN was trained for feature extraction on red-free RNFL photographs and SD-OCT images and a support vector machine model was trained as a regression model | Predicted mGCIPL thickness from red-free RNFL photographs and classified as glaucomatous, suspect, or normal eyes | The mGCIPL thickness measurement on SD-OCT | 789 pairs of RNFLPs / 431 eyes / 259 participants (183 eyes of 114 healthy controls, 68 eyes of 46 glaucoma suspects, and 180 eyes of 99 glaucoma patients) | Normal: Female (56.5%)  Glaucoma suspect: Female (62.7%)  Glaucoma: Female (58.3%) | The HDLM’s ability to differentiate eyes with glaucomatous VF loss from healthy eyes: AUC = 0.918, 95% CI: 0.898–0.939; sensitivity 0.901, 95% CI: 0.852–0.945; specificity 0.805, 95% CI: 0.764–0.843. The classifier’s classification accuracy for the three groups (normal, glaucoma suspect, glaucoma) was 81.7% |
